# Engineering Polymer-Based Porous Membrane for Sustainable Lithium-Ion Battery Separators

**DOI:** 10.3390/polym15183690

**Published:** 2023-09-07

**Authors:** Lei Li, Yutian Duan

**Affiliations:** 1 SINOPEC Nanjing Research Institute of Chemical Industry Co., Ltd., Nanjing 210048, China; 2 College of Electrical Engineering, Zhejiang University, Hangzhou 310027, China

**Keywords:** lithium-ion battery separator, porous membrane, polymer, polyethylene, polypropylene, poly(vinylidene fluoride)

## Abstract

Due to the growing demand for eco-friendly products, lithium-ion batteries (LIBs) have gained widespread attention as an energy storage solution. With the global demand for clean and sustainable energy, the social, economic, and environmental significance of LIBs is becoming more widely recognized. LIBs are composed of cathode and anode electrodes, electrolytes, and separators. Notably, the separator, a pivotal and indispensable component in LIBs that primarily consists of a porous membrane material, warrants significant research attention. Researchers have thus endeavored to develop innovative systems that enhance separator performance, fortify security measures, and address prevailing limitations. Herein, this review aims to furnish researchers with comprehensive content on battery separator membranes, encompassing performance requirements, functional parameters, manufacturing protocols, scientific progress, and overall performance evaluations. Specifically, it investigates the latest breakthroughs in porous membrane design, fabrication, modification, and optimization that employ various commonly used or emerging polymeric materials. Furthermore, the article offers insights into the future trajectory of polymer-based composite membranes for LIB applications and prospective challenges awaiting scientific exploration. The robust and durable membranes developed have shown superior efficacy across diverse applications. Consequently, these proposed concepts pave the way for a circular economy that curtails waste materials, lowers process costs, and mitigates the environmental footprint.

## 1. Introduction

Owing to the increasing demand for environmentally-friendly products, LIBs have received widespread attention as an energy storage solution. LIBs are widely considered one of the most sustainable and powerful energy storage technologies available today and are commonly used in industries such as mobile devices, electric vehicles (EVs), and energy storage systems [1,2]. Given the escalating global demand for clean energy and sustainable progress, the social and economic significance of LIBs continues to gain momentum [3,4,5,6]. Due to their remarkable energy density, prolonged storage life, wide operational temperature range, and elevated battery voltage, LIBs have emerged as the predominant contender in the realm of energy storage batteries, finding widespread utility in various domains such as aerospace, artificial satellites, and efficient energy storage for both military and civilian electrical appliances [7]. Additionally, in the current low-carbon global environment, new energy sources have assumed prime importance in the global agenda, specifically with high-capacity LIBs serving as a key power source for 21st-century new-energy EVs [5,8,9]. Notably, as the EV industry and the electronic information sector (encompassing mobile phones, electric tools, and digital cameras) continue to flourish, the demand for LIBs will persistently burgeon [1,10,11]. Therefore, the concept of LIBs has promising prospects, and higher requirements have been put forward for LIB performance, particularly in terms of electrochemical performance (e.g., energy density) and safety [3,12].

**Figure 1:**
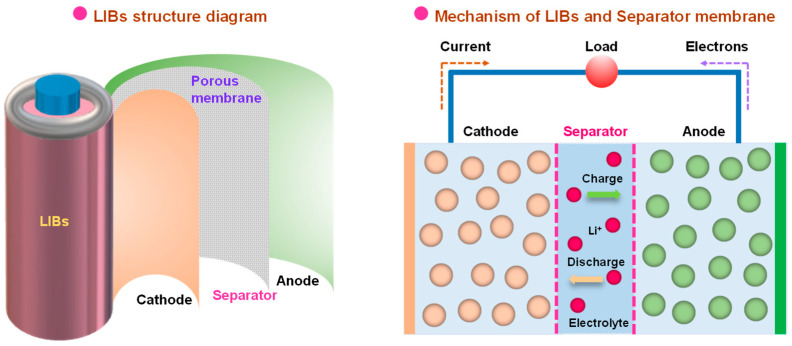
Schematic illustration of rechargeable LIBs consisting of cathode and anode electrodes, electrolytes, and porous membrane-based separators.

LIBs are mainly composed of positive (cathode) and negative (anode) electrodes [13,14], electrolytes, and separators [15,16,17,18,19], wherein the separator, mainly consisting of a porous membrane material, assumes an indispensable and critical role within LIBs (Figure 1). The porous membrane absorbs electrolytes and is assembled between the battery cathode and anode electrodes, which is a crucial section in LIB separators [9,20]. Throughout the charging and discharging cycles of LIBs, lithium ions (Li^+^) migrate between the cathode and anode electrodes through a separator and, thus, conduct electricity [21]. Specifically, a separator membrane fulfills several vital functions within an LIB. First, it serves to maintain a physical barrier between the cathode and the anode, preventing short circuits arising from direct electrode contact [22]. Second, the presence of micropores in the membrane allows for the passage of Li^+^, forming a seamless charging and discharging circuit [23]. Furthermore, the membrane exhibits the ability to shut down in response to appropriate temperatures, subsequently closing the micropores, thereby serving as a protective open circuit that safeguards against fires or explosions resulting from thermal runaway caused by elevated temperatures and short circuits. Thus, it offers a protective shield for both battery users and equipment [24,25]. It is worth mentioning that the separator material, typically a thin, polymer-based porous membrane, may be inherently insulating. However, its structural composition and performance characteristics significantly influence the overall performance of LIBs. The basic performance parameters of the membrane mainly encompass thickness, mechanical strength, porosity, wettability, thermal shrinkage, electrochemical stability, and so on [26,27,28]. Therefore, appropriate material selection and structural design of separators determine these performance parameters and play a crucial role in ensuring the safe utilization and long-lasting charging and discharging cycles of LIBs.

Currently, the most commonly utilized polymeric materials for producing porous membranes in rechargeable batteries, particularly LIBs, include polyethylene (PE), polypropylene (PP), poly(tetrafluoroethylene) (PTFE), poly(vinylidene fluoride) (PVDF), poly(methyl methacrylate) (PMMA), polyimide (PI), polyesters, poly(vinyl chloride) (PVC), poly(ethylene oxide) (PEO), polyacrylonitrile (PAN), poly(ethylene terephthalate) (PET), and cellulose and its derivatives [29,30]. Microporous polyolefin membranes, featuring PE, PP, and their blends, hold prominence in the commercial market as separators for secondary rechargeable batteries utilizing liquid electrolytes, including LIB, due to their superior mechanical strength, chemical and electrochemical stability, cost-effective production, tunable pore sizes, thermal shutdown capability, and reasonable material costs [30,31,32]. Notably, ultra-high molecular weight polyethylene (UHMWPE) plays a crucial role in lithium battery separator materials and is highly applied in the global automotive battery market [7,33,34]. Moreover, the UHMWPE membrane provides excellent safety protection for overcharging, short circuit, and explosions when the temperature rises, thus rendering it remarkably suitable for high-efficiency and high-power batteries. At elevated temperatures, the UHMWPE melts smoothly, transitioning into a rubber-like gel without mobility due to the highly entangled molecular chain, thereby impeding undesired membrane collapse [35,36,37]. Preparation methods for polyolefin microporous membranes for LIB separators mainly include the dry method (i.e., melt extrusion stretching method) and the wet method (i.e., thermally induced phase separation method, TIPS) [30,38,39,40,41]. These membranes are created through sequential biaxial stretching, renowned for their remarkable mechanical robustness and consistent pore size distribution [42]. However, these separators manifest noteworthy thermal contraction and exhibit a limited affinity for electrolytes due to their electrolyte-phobic nature and poor electrolyte retention during cycling processes [29,31,43]. These factors compromise their suitability for application in high-power, high-capacity, and high-temperature LIBs. Consequently, over the past decade, numerous researchers have endeavored to address the limitations of conventionally used separators by modifying their structural and material makeup, thereby amplifying the versatility of LIBs across a diverse range of applications, particularly those with elevated power requirements [34,44,45,46].

Notably, the utilization of sustainable electrochemical energy storage devices holds numerous benefits in fostering both a circular and green economy [1,9,10,11,38]. As one of the economically applicable directions for clean energy, the main focus of LIB separators is to develop and prepare new membranes for high-performance batteries [8]. Herein, this review highlights the significance of porous membrane for separators in LIBs, including the fundamental prerequisites and performance benchmarks of ideal separators, encompassing the chemical, mechanical, electrical, and electrochemical properties of porous separator membranes for batteries. Additionally, this review investigates cutting-edge advancements in the preparation, design, modification, and optimization of porous membranes, leveraging polymeric materials including but not limited to PE, UHMWPE, PP, and PVDF. Furthermore, the future prospects of polymer-based composite membranes for LIB applications and the prospective concerns of researchers are outlined. The overall objective of this review is to assist relevant researchers in comprehending the underlying design principles, strategies, methodologies, and potential application scenarios associated with porous membranes for LIB separators. Overall, the development of robust, efficient, and innovative LIB separators plays a pivotal role in realizing the tenets of a circular economy, as well as the broader objectives of resource rationalization and environmental sustainability [47].

## 2. Characteristics Outline of LIB Separators and Polymeric Membranes

### 2.1. Requirements and Features of LIB Separators

**Figure 2:**
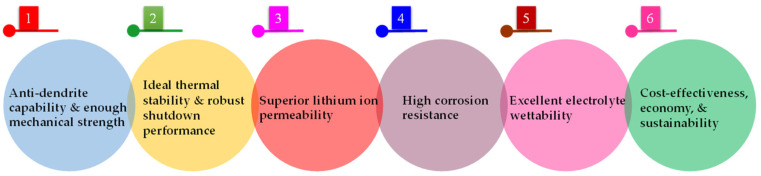
Schematic illustration of separator performance requirements to consider when selecting the appropriate separator for rechargeable LIBs.

The separator serves as a critical internal component within the structure of LIBs, which determines the battery interface composition and internal resistance, thereby directly affecting battery efficiency, discharge capacity, cycling performance, safety, and so on [48,49]. Positioned between the cathode and the anode, the LIB separator plays a critical role in preventing short circuits resulting from direct contact between the cathode and the anode active material, while allowing the rapid transfer of Li^+^ during charge and discharge processes [50,51,52,53,54]. Considering that the separator material is a non-conductive material responsible for preserving electrolytes crucial for establishing ion transfer pathways amidst intricate electrochemical processes, its physical and chemical attributes pose a considerable impact on battery performance (Figure 2). In general, utilizing a thinner and more porous separator results in a reduction in internal resistance, thereby improving discharge capacity [55,56]. However, high porosity and low thickness can compromise the mechanical strength of the separator, thus necessitating the development of materials that balance electrochemical and mechanical properties to improve overall battery performance [57,58]. Specifically, the commercialization of polymeric electrolytes for LIBs has recently been developed in recent years, serving as both channels for ion migration and separators between electrode materials [59,60,61,62,63]. Notably, polymer electrolytes (PEs) have attracted tremendous interest in next-generation LIBs due to their high safety and energy density, along with their superior flexibility [64,65]. Compared to liquid electrolytes (LEs), PEs offer a range of advantages, such as mechanical strength, tunability, and easy processability, making them a promising option for flexible and wearable electronic devices [66]. Furthermore, among the various types of solid electrolytes (SEs), PEs boast benefits encompassing low flammability, high flexibility, exceptional thermal stability, and ideal safety features [67]. Hence, diverse electrolytes necessitate the rational design and selection of appropriate separators for LIBs.

#### 2.1.1. Anti-Dendrite Capability and Enough Mechanical Strength

Notably, lithium dendrites could form during long-term charge/discharge cycles, presenting the most prevalent and hazardous causes of internal short circuits in LIBs [68]. Ultimately, these dendrites may impact the separator upon reaching a specific magnitude, fostering pressure and needle-like protrusions that give rise to mechanical malfunction and a consequent short circuit between the cathode and anode electrodes, posing substantial safety hazards [69]. Moreover, during the battery assembly process, bending or tilting may result in electrode contact, leading to internal short circuits. Note that a thicker separator provides improved mechanical durability, reducing the incidence of short circuits during assembly. In general, separators for LIBs are typically less than 25 μm in thickness. For high-energy batteries, a single-layer membrane with a thickness of 20 μm or 16 μm is most commonly used [70]. However, electric and hybrid vehicles require a thicker separator of 40 μm in order to achieve high battery capacity and elevated current discharge. Herein, achieving a certain level of mechanical strength with sufficient puncture and tensile strength is imperative, and this can be accomplished by utilizing a thinner separator with higher porosity, which in turn decreases the internal resistance of the battery and improves high-rate discharge capabilities [71].

#### 2.1.2. Ideal Thermal Stability and Robust Shutdown Performance

**Figure 3:**
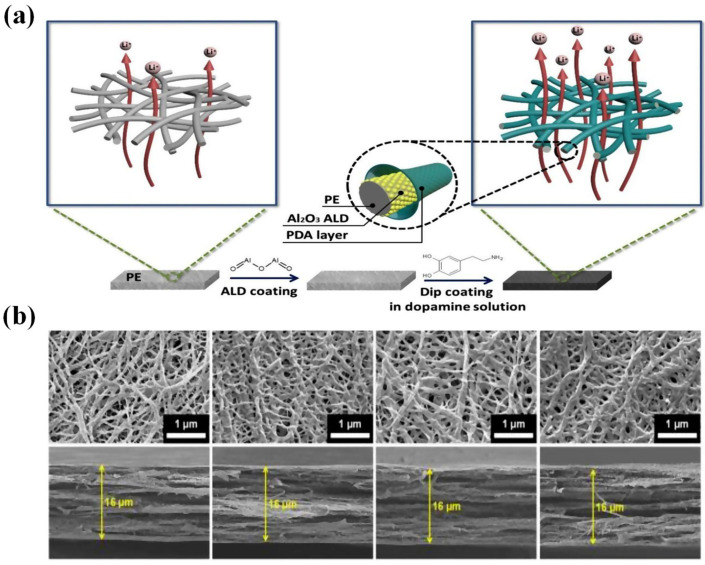
(**a**) Schematic illustration of the preparation of the PE/Al_2_O_3_/PDA separator by synergistic inorganic and organic coatings through atomic layer deposition and PDA treatment. (**b**) SEM images and corresponding cross-sectional views of bare PE, PE/Al_2_O_3_, PE/PDA, and PE/Al_2_O_3_/PDA separators. Reprinted with permission from reference [84], copyright Elsevier 2019.

LIBs inevitably generate heat during charge and discharge; in particular, when there is a short circuit or overcharging, the heat released is considerably increased [72,73]. To adapt to temperature changes and improve thermal stability in normal working environments, the separator should maintain excellent dimensional stability and mechanical strength within the temperature range of −20 °C to 90 °C [24]. To improve thermal stability, current development is focused on coated (or composite) separators that can provide significant advantages, especially improving the thermal stability of the separator [74]. By coating, membrane heat and puncture resistance can be significantly boosted, thereby reducing safety concerns related to rapid charging and heat dissipation, as well as thermal shrinkage of the membrane (leading to contact between anode and cathode), combustion, and explosion [75]. Currently, there are two prevailing coating types available, i.e., inorganic coatings and organic coatings [76,77,78]. Inorganic coating generally refers to the composite modification on the separator membrane with a layer of ceramic particles to enhance their comprehensive performance [79]. Herein, the inorganic ceramic layers are composed of inorganic particles and adhesives, including but not limited to aluminum oxide (Al_2_O_3_), boehmite, zirconium dioxide (ZrO_2_), silicon dioxide (SiO_2_), and titanium dioxide (TiO_2_), among others [80,81]. Compared to organic coatings, inorganic composite membranes exhibit remarkable mechanical strength, exceptional stability, superior thermal resistance, and minimal shrinkage, which can prevent thermal runaway and significantly improve the stability of LIBs [82]. Moreover, they offer numerous advantages, including mature technology, high cost-effectiveness, and significant economic benefits. Correspondingly, organic coating, also known as organic composite modification, entails the coating of an organic substance (e.g., PVDF, PAN, PMMA, polydopamine (PDA), aramid, and their composites) onto the surface of the separator membrane, which exhibits remarkable liquid absorption and retention capabilities, along with high adhesion properties [7]. Moreover, the ideal compatibility of organic compounds with the electrolyte can significantly diminish the contact angle between the modified separator and the electrolyte, thereby improving electrochemical performance (i.e., ion conductivity, cycle life, rate capability, and so on) [83]. Notably, the synergistic integration of organic and inorganic coatings can optimize the aforementioned benefits, enhance the efficiency of high-power rapid charging and discharging, and bolster the safety of LIBs (Figure 3) [84].

Notably, thermal shutdown is another crucial aspect of separator performance, resulting in the closure of the micropores once the battery is short-circuited and the temperature is abnormal beyond the operating range, which blocks the continuous transmission of ions and avoids thermal runaway, thus safeguarding the batteries [29]. The shutdown mechanism is achieved through the combination of two substances with different melting temperatures (T_m_). Due to the thermoplastic properties of polymeric materials, the porous ion-conducting membrane can transform into an insulating layer without pores as the temperature approaches T_m_. Upon reaching the T_m_ of the fusible substance, it melts, blocking pores, while the high T_m_ substance maintains structural integrity [85]. The safety window temperature in the shutdown mechanism represents the T_m_ differential between the two substances. In addition, the mechanical integrity of the separator should not be affected by the shutdown process, otherwise the cathode/anode electrodes may come into direct contact, causing chemical reactions that could lead to thermal runaway [86]. For example, the shutdown and T_m_ of the PE–PP double-layer separator for rechargeable batteries are ~130 °C and ~165 °C, respectively [25]. Furthermore, a fusible substance with a lower T_m_ ensures a greater thermal sensitivity of the membrane, larger safety windows, and superior safety performance of the membrane [24].

#### 2.1.3. Superior Lithium Ion Permeability

Separator materials must facilitate ion mobility, provide ample and unobstructed pathways for Li^+^ flow, and function as an electron insulator to uphold insulation between the anode and cathode. The permeability of the separator is a crucial characteristic that can be quantified using the MacMullin number [30]. Notably, the MacMullin number is directly related to air permeability, which is often expressed as the Gurley value, i.e., the time for air to traverse a unit area of the separator at a fixed pressure [87]. The Gurley value is contingent upon the tortuosity of the pores, as well as the thickness and porosity of the separators, wherein a lower Gurley value suggests increased porosity and diminished tortuosity [42]. Moreover, homogenous permeability is critical to achieving a prolonged cycle life, considering that any variation in the permeability pattern can result in a nonuniform distribution of current density and dendrite formation on electrode surfaces. Additionally, to promote effective Li^+^ transmission during charge and discharge, a sufficient porosity is required [30], with commonly used separator apertures in the market being less than 1 μm. Meanwhile, to enhance safety during usage, porosity is generally recommended not to exceed 50%.

#### 2.1.4. High Corrosion Resistance

The chemical stability of a separator refers to its ability to withstand chemical reactions with electrolytes and electrode materials [88]. Considering that LIB separators operate in an environment surrounded by electrolytes and active electrodes, the separator must maintain long-term stability even under strong oxidation and reduction conditions, and avoid reacting with electrolyte and electrode materials to ensure the safety of LIBs [89]. Notably, separator resistance to electrolyte corrosion could be qualitatively or quantitatively measured to assess the separator chemical stability [90,91]. Given that various LIBs require a separator that is resistant to organic solvents, high-strength polyolefin-based separators are commonly employed [92,93], unlike polymers with labile bonds, such as polycarbonates, polyesters, and polypeptides [94,95], which are easily decomposed by chemical or enzymatic factors [96]. Additionally, inorganic reinforcing nanomaterials are typically added to construct more stable and robust separators, encompassing Al_2_O_3_, SiO_2_, TiO_2_, ZrO_2_, and so on [29]. Due to its exceptional stability and strong affinity for electrolytes, Al_2_O_3_ is widely regarded as a preferred reinforcement material [97]. For example, Al_2_O_3_-modified PE separators in LIBs demonstrated enhanced safety and reliability, delivering excellent cycling performance and stability at 55 °C and exceptional thermal safety even at 200 °C [97]. Currently, SiO_2_ has also been utilized to modify separators for enhanced stability and electrolyte affinity, which are ideal materials for supporting the separator matrix for LIBs due to high polarity and exceptional thermal stability [98]. For instance, the inclusion of SiO_2_ can enhance critical properties of UHMWPE separators, including stability, electrolyte wettability, and ionic conductivity. As such, the resulting LIB displayed superior discharge capacities of 165 mAh g^−1^ at 0.1 C-rate and 123 mAh g^−1^ at 5 C-rate, alongside ideal cycling performance over 100 cycles [99]. Meanwhile, TiO_2_ nanoparticles have been extensively explored for LIB separator modification due to their high hydrophilicity, superior chemical and thermal stability [100,101]. Here, a TiO_2_-grafted PE separator was developed that not only demonstrated significantly enhanced stability even at 150 °C, but also superior electrochemical performance compared to bare PE separators, thus presenting a highly advantageous option for the construction of safer LIBs [102]. Additionally, ZrO_2_ is also renowned for its exceptional chemical and thermal stability compared to other ceramic materials such as Al_2_O_3_, SiO_2_, and TiO_2_ [103]. When added to separators, it could boost Li^+^ transference number and ionic conductivity while simultaneously enhancing interfacial stability between electrodes and electrolytes. A LIB featuring the ZrO_2_-modified PE separator exhibited exceptional cyclic performance with a remarkable retention rate (96.2%) after 50 cycles, and stability was considerably enhanced even after operation at 200 °C [104]. Moreover, other ceramic particles encompassing cerium dioxide (CeO_2_), calcium phosphate (Ca_3_(PO_4_)_2_), magnesium oxide (MgO), and aluminum oxyhydroxide (AlOOH) have also displayed promising functionality for stable separators with exceptional comprehensive properties when skillfully engineered [7].

#### 2.1.5. Excellent Electrolyte Wettability

Wettability refers to the capacity to efficiently absorb and retain electrolytes without experiencing elongation or contraction [105]. Note that, if the separator can be quickly and thoroughly penetrated by electrolytes, the internal resistance of LIBs can be significantly reduced, thereby enhancing ion conductivity and charge and discharge capacity [106]. The primary function of the LIB electrolyte is to conduct ions between cathode and anode, serving as a medium for charge and discharge [107]. Poor wettability increases resistance of the separator, affecting cycle performance and charge/discharge efficiency [108,109]. Hence, it is necessary to modify the surface of the membranes to be more hydrophilic. Common methods involve surface or pore functionalization with hydrophilic groups or surfactant treatment [110,111]. It should be noted, however, that surfactants may be lost through electrolyte washing during cycling and storage, resulting in temporary hydrophobicity. Conversely, hydrophilic functional groups can be grafted onto membrane surfaces through thermal spray by carefully selecting appropriate gases, resulting in improved durability, encompassing hydroxyl, carbonyl, carboxyl, amino, and sulfonyl groups [32]. Additionally, wettability can also be increased through sulfonation, fluorination, graft polymerization, etc. [112]. Contact angle testing and electrolyte absorption are widely used as characterization methods to measure wettability. The methodology entails dispensing electrolyte onto the separator and subsequently measuring the contact angle (θ). A lower θ value indicates superior wettability of the separator surface towards the electrolyte [14].

#### 2.1.6. Cost-Effectiveness, Economy, and Sustainability

Developing a cost-effective production process for separators is crucial for cost reduction. Thus, reducing the thickness of separators is an efficient way to cut down expenses driven by the lowered prices of raw materials [10,113]. Moreover, the emergence of the LIB and separator industries has resulted in a significant shift from dependence on petroleum resource endowments to reliance on manufacturing endowments and recycling of lithium batteries, thereby enabling sustainable development through a circular economy [114]. Specifically, with the growing global concern for environmental protection and sustainable energy development, LIBs hold great value as an energy storage solution in fields like EVs, mobile devices, and energy storage systems [115,116]. With the transformation of energy structures in various countries and the increasing deployment of renewable energy, the LIB and separator industries have emerged as one of the most promising energy industries with the surging worldwide demand for sustainable development and clean energy [117]. Therefore, the preparation and design of separators and their components should be consistent with the worldwide concepts of cost-effectiveness, sustainability, and ecological consciousness.

### 2.2. Physical and Chemical Properties of Separator Membranes

**Figure 4:**
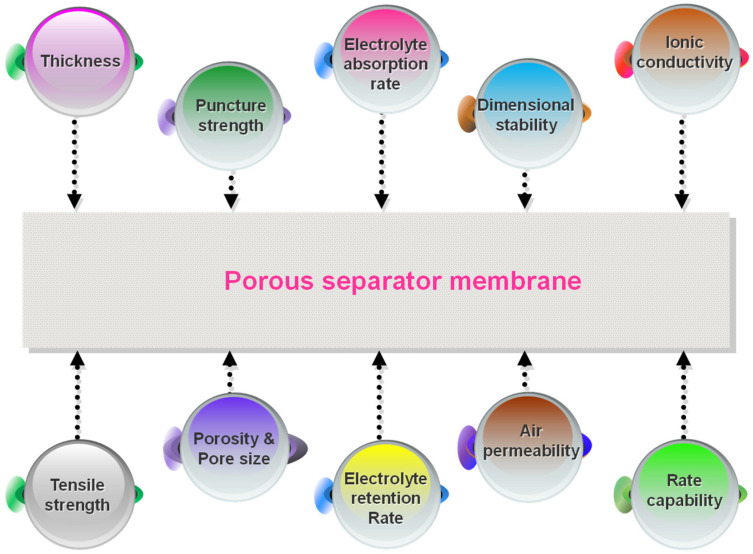
Schematic illustration of the physical and chemical properties to consider when designing appropriate separator membranes for rechargeable LIBs.

The separator is a passive component in LIBs and plays a critical role in enhancing battery performance and safety in practical applications. The various performance indicators of the membrane are interrelated and significantly impact the overall performance of the battery. Various techniques and methods are utilized to characterize membrane performance, encompassing tensile strength, puncture resistance, thermal stability, porosity, electrolyte wettability, chemical stability, and so on [23,31]. Due to its implementation in various large-scale types of equipment such as EVs or energy storage stations, LIBs require superior mechanical strength, capacity, thermal stability, rate capability, excellent electrochemical performance, and prolonged cycle performance. Accordingly, an optimal separator is essential to meet certain critical physical and chemical properties (Figure 4).

#### 2.2.1. Thickness

The membrane thickness should be kept to a minimum in applications with high-energy density and high-power scenarios. However, this can jeopardize the battery’s safety and mechanical durability. The thinner the membrane, the lower the mechanical strength, increasing the risk of internal short circuits during long-term use due to perforation and battery assembly [24]. Conversely, thicker separators could raise resistance, thus lowering the electrochemical performance of the battery. Moreover, thickness uniformity is critical because changes in thickness can hasten battery deterioration and shorten its lifespan, necessitating a consistent current distribution throughout battery operation [118]. Therefore, it is essential to reduce thickness and volume to boost the total energy density of the battery without sacrificing the minimum tensile strength of the membrane, preventing cracking both during assembly and throughout the battery service life. Generally, to ensure stable and effective charging and discharging cycles and normal battery usage, it is ideal to appropriately control the thickness of the separator within 25 μm [29].

#### 2.2.2. Tensile Strength

The membrane tensile strength is dictated by its material and manufacturing process, primarily characterized by its strength in the mechanical direction (MD) and transverse direction (TD). In order to prevent damage during transportation and assembly, as well as battery short circuits caused by lithium dendrite growth, the membrane requires a minimum tensile strength of 20 MPa [119]. Moreover, the separator must not experience significant elongation under tension to prevent width shrinkage. Generally, higher porosity results in lower impedance but weaker strength. Notably, uniaxial stretching can cause a discrepancy in strength between stretching and vertical directions, whereas biaxial stretching results in consistent strength in both directions [28]. The ASTM D882-10 standard [120] specifies test parameters, including fixture spacing, stretch rate, and sample size, for assessing membrane tensile strength [30]. According to ASTM D882, the minimum tensile strength of separators with a thickness of 25 µm is 98.06 MPa [71].

#### 2.2.3. Puncture Resistance

Puncture resistance refers to the mass applied to a specific needle to puncture a given membrane sample, which is used to characterize the susceptibility of the membrane to short circuits during processes such as assembly [121]. Considering factors such as curling and packaging during the production of the membrane, assembly and disassembly of the battery, and repeated charging and discharging, the membrane must have a certain level of physical strength to withstand damage from puncture, physical impact, compression, and wear. Therefore, the puncture strength of the membrane should be investigated, and the membrane must have a certain strength of puncture resistance to prevent short circuits. Based on ASTM D3763 [122], the puncture resistance of the separator must meet or exceed 300 g mil^−1^ [106]. Specifically, the penetration strength of bare PE and PE–PVDF separators are of 5.67 and 6.20 N, respectively [123].

#### 2.2.4. Porosity and Pore Size

Porosity refers to the ratio of the pore volume to the total volume of the separator membrane, which can be calculated as a percentage using the following Equation (1). The LIB separator should have sufficient porosity to absorb the electrolyte and maintain high conductivity [14]. Notably, optimal porosity can ensure that sufficient electrolyte is captured within the membrane pores to maintain elevated ionic conductivity, while membranes with low porosity retain fewer electrolytes, resulting in increased internal resistance, and excessive porosity could compromise the thermal shutdown of the battery [124,125]. Meanwhile, a relatively uniform pore size is a prerequisite to avoid electrode performance degradation caused by uneven current [126,127]. The membrane pores should have a uniform shape with appropriate size to allow Li^+^ to pass through the absorbed electrolyte and prevent internal short circuits. Notably, appropriately increasing porosity while maintaining a certain degree of mechanical and thermal stability is the trend of LIB separators [7]. The current mainstream product of LIB separators has a porosity of 40% with a pore size on the submicron scale (especially with a membrane thickness of 25 μm). Specifically, the pore size of commercial membranes typically ranges from 0.03 to 0.12 μm and exhibits a narrow and uniform distribution [128]. Moreover, the maximum pore size should not exceed the average pore size distribution by more than 0.01 μm [129].

(1) $$\mathrm{Porosity}\;(\%)\;=(1-\frac{\rho_{M}}{\rho_{P}})\;\times \;100$$

where *ρ_M_* and *ρ_P_* represent the actual density of the separator membrane and the polymer, respectively.

#### 2.2.5. Electrolyte Absorption and Retention Rate

Obviously, the separator should be able to absorb enough electrolyte and maintain absorption during battery operation, which could improve LIBs performance by reducing separator resistance and enhancing charge and discharge efficiency. The wetting rate is related to the electrolyte filling time, as well as the polymer type, pore size, porosity, and curvature of the membrane [105,111]. Note that the electrolyte should thoroughly wet the membrane to reduce the internal resistance of the battery. In addition, the separator must be quickly immersed in electrolyte to facilitate battery assembly during large-scale production. Note that electrolyte absorption by the separator depends on the hydrophilicity and crystallinity of the polymer matrix, as well as the porosity of the membrane [112]. Although electrolyte absorption can cause a certain loss of mechanical strength, the active substances of adjacent electrodes will not be completely utilized if the electrolyte is not distributed thoroughly and uniformly within the membrane. Equations (2) and (3) are used to calculate the electrolyte absorption rate and retention rate of the separator [29]:

(2) $$\mathrm{Electrolyte}\;\mathrm{absorption}\;\mathrm{rate}\;(\%)=\frac{W{-\;W}_{0}}{W_{0}}\;\times \;100$$

(3) $$\mathrm{Electrolyte}\;\mathrm{retention}\;\mathrm{rate}\;(\%)=\frac{W\;{-\;W}_{0}}{W}\;\times \;100$$

where *W*_0_ is the original mass of the separator and *W* is the mass of the separator after immersing into the electrolyte for 1 h.

#### 2.2.6. Dimensional Stability

The membrane must maintain a flat surface and avoid curling when placed and immersed in the electrolyte. However, in practical battery assembly, incomplete contact may occur unexpectedly between the electrode and the membrane, resulting in internal short circuits. Moreover, batteries emit thermal energy when undergoing both charging and discharging processes, particularly when experiencing short circuits or being overcharged, which leads to the release of a substantial amount of heat. Therefore, it is imperative for the membrane to uphold its initial dimensional stability and mechanical resilience, functioning as a partition to separate the cathode and anode while averting any potential short circuits, particularly as the temperature escalates [130]. Herein, the separator must maintain its size and avoid significant shrinkage even at high temperatures during battery operation. Note that the thermal shrinkage of the separator in both the MD and TD directions should be less than 5% (in vacuum) after holding at 90 °C for 60 min [131]. The thermal stability of the separator was evaluated by measuring the two-dimensional change of square separators (2 × 2 cm^2^ sample) after heat treatment in the temperature range of 90 to 145 °C for 30 min. The thermal shrinkage rate was then calculated using Equation (4):

(4) $$\mathrm{Thermal}\;\mathrm{shrinkage}\;(\%)=\frac{S_{0}-S}{S_{0}}\;\times \;100$$

where *S*_0_ is the original area of the separator and *S* is the area of the separator after the heat treatment.

#### 2.2.7. Air Permeability

The battery separator permeability is typically characterized using the Gurley value, in accordance with ASTM D726-94 [106,132]. This measurement is based on air permeability and expresses the time required for a specific volume of air to pass through a defined separator area under specific pressure conditions. The Gurley value provides an indication of the battery internal resistance, with higher values indicating greater resistance. The Gurley value is influenced by the separator porosity, fraction of open pores, and the tortuosity [133]. The consistent permeability of the separator eliminates discrepancies in current distribution, thus preventing dendrite formation and contributing to the extended cycle life of LIBs. The ideal Gurley values of LIB separator should be below 0.025 s µm^−1^ [29]. The Gurley value (*G*) can be calculated using Equation (5) [57]:

(5) $$G=\frac{\eta_{\mathrm{air}}\mathrm{VL}}{k\Delta \mathrm{PA}}$$

where *η_air_* represents air viscosity, *V* denotes air volume, *L* refers to separator thickness, *k* refers to permeability, Δ*P* represents pressure difference, and *A* represents the area of the separator.

#### 2.2.8. Electrochemical Performance

The electrochemical performance of battery separators mainly includes various factors such as electrochemical stability, ion conductivity, cycling stability, rate capability, etc. [134,135,136]. For the sake of ensuring the longevity of batteries, it is crucial for the separator to possess exceptional chemical stability with respect to the electrolyte, overcoming the formation of unnecessary degradation and mechanical strength loss during charging and discharging. Moreover, separators must have stable electrochemical properties in strong oxidation and reduction environments composed of active electrode components, and should not create impurities that may impact battery operation [137]. Notably, ion conductivity with liquid electrolytes (usually 10^−1^–10 mS cm^−1^) is heavily associated with the membrane’s ability to transport Li^+^ while being immersed in the electrolyte [138]. Thus, superlative ion conductivity and rate capability necessitate the separator to have exceptional electrolyte wettability. However, flammable organic solvents in liquid electrolytes such as ethylene carbonate and propylene carbonate pose significant safety concerns [139,140]. The ion conductivity can be measured using electrochemical impedance spectroscopy (EIS) with the membrane sample soaked in the electrolyte, then calculated based on the measured volume resistance, thickness, and area of the separated sample [141]. Also, the cycling stability and rate capability of the battery separator are both examined by assembling the electrolyte-soaked separator between two symmetrical lithium electrodes, and the initial LIBs should display high cycling stability and capability [136,142]. Note that superb electrolyte wettability can enhance cycling stability, improve capacity retention, and battery efficiency. This, however, depends on whether the separator is convenient for Li^+^ transportation and whether it can display excellent interface compatibility between the electrode and separator [143,144].

The ionic conductivity was analyzed by measuring the EIS of LIBs with two stainless steel plates as electrodes with an amplitude of 10 mV at 25 °C, and the frequency range is from 0.1 Hz to 105 Hz [23]. The ionic conductivity can be calculated with Equation (6):

(6) $$\sigma \;=\frac{d}{R_{b}S}$$

where *σ* is the conductivity, *d* is the thickness of the separator, *R_b_* is the bulk resistance, and *S* is the area of the contact.

For rate capability of Li^+^ transfer at the solid−liquid interface, the core of electrochemical kinetics is related to the potential difference at the solid−liquid interface through the Butler−Volmer Equation (7) [134]:

(7) $$J_{r}=2\mathrm{ka}c_{e}^{0.5}c_{s,\mathrm{surf}}^{0.5}{\left(c_{s,\max}-c_{s,\mathrm{surf}}\right)}^{0.5}\sin h\left[\frac{0.5F}{\mathrm{RT}}(\Phi_{s}{-\Phi }_{l}{-V}_{\mathrm{ocv}})\right]$$

where *J_r_* represents the ionic flux for the interfacial reaction, *k* denotes the reaction rate constant, *a* refers to the surface area to volume ratio of the particle, *c_e_* represents the Li^+^ concentration in the electrolyte adjacent to the particle surface, *c_s,max_* represents the maximum Li^+^ concentration within the particle, *c_s,surf_* designates the Li^+^ concentration at the particle surface, *F* signifies the Faraday constant, *R* stands for the gas constant, *T* indicates the temperature, *Φ_s_* denotes the solid potential, *Φ_l_* stands for the electrolyte potential, and *V_ocv_* represents the open-circuit potential.

## 3. Manufacturing: Dry and Wet Methods Based on Uniaxial/Biaxial Stretching

### 3.1. Uniaxial/Biaxial Stretching of Separator Membrane

**Figure 5:**
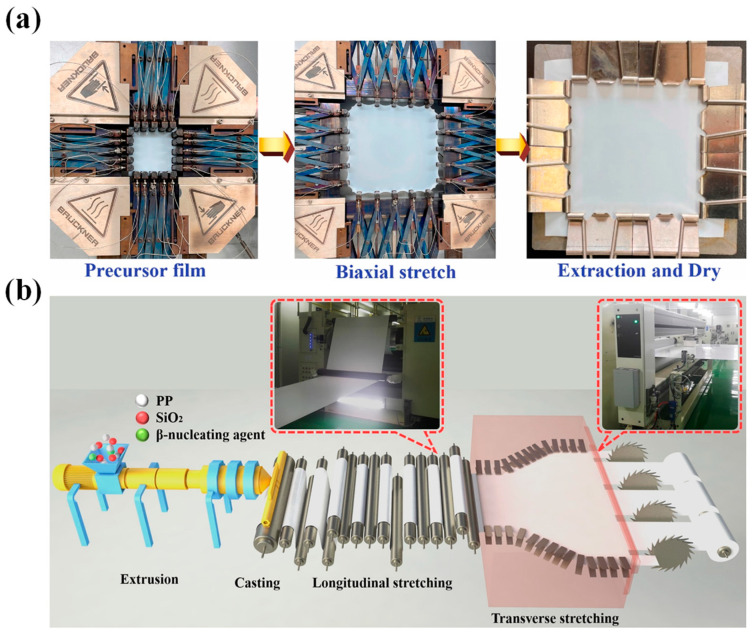
(**a**) Schematic illustration of the fabrication process for a microporous membrane by simultaneous biaxial stretching, extraction, and drying based on the wet method. Reprinted with permission from reference [154], copyright Elsevier 2021. (**b**) Schematic illustration of the casting and sequential biaxial stretching processes based on the dry method. Reprinted with permission from reference [155], copyright Elsevier 2022.

LIB separator membranes can be categorized into several types, encompassing woven or non-woven membranes, microporous membranes, composite membranes, cellulose-based membranes, and electrolyte membranes [34,109,125]. Polyolefin materials such as PE and PP are preferred due to their exceptional mechanical properties, chemical stability, mature manufacturing processes, and low cost; thus, they have been frequently employed as separator components in LIBs research and development [28,34]. While some recent investigations have delved into the utilization of substitute materials (e.g., PVDF) [145,146,147,148], as the primary polymer in phase inversion methods and cellulose-based composite membranes in LIBs [83,102,149,150,151,152,153], commercial polyolefin-based LIB separators still predominated the overall LIBs markets, encompassing PE, PP, and UHMWPE [7,34]. Preparation procedures can be segregated into dry and wet methods, based on the varying formation mechanisms of microporous structures [30,34] (Figure 5).

**Figure 6:**
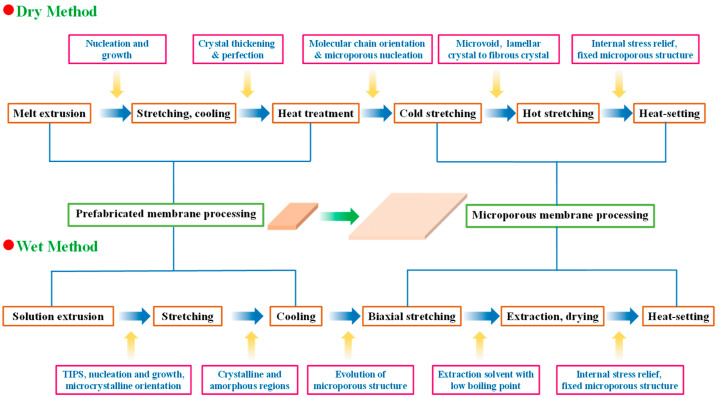
Schematic illustration of the process flow and corresponding structural evolution of LIB separator membranes based on dry and wet methods.

Industrially, the manufacturing focus has been on wet-processed biaxially stretched PE membranes and dry-processed uniaxially stretched PP membranes. Dry stretching can be categorized into uniaxial and biaxial stretching, while wet stretching can be divided into synchronous and asynchronous stretching (Figure 6). Note that dry biaxial stretching relies on the density difference between various phases of PP to create crystal transformation and form microporous membranes. Correspondingly, the wet method, also known as TIPS, utilizes temperature variations to induce phase separation [156]. It is noteworthy that biaxial stretching of PP or PE membranes can be achieved either simultaneously or in sequence. Specifically, sequential biaxial stretching involves stretching the casting membrane in the MD direction while gradually increasing the roller speed. Next, the stretcher frame is stretched in the TD direction with the membrane clamped on each side by fasteners connected to the moving chain. The stretched membrane then passes through the heating zone in the oven [58]. Alternatively, simultaneous biaxial stretching can be utilized, wherein the cast or blown membrane is stretched in both MD and TD directions synchronously in a single process, thus preventing scratches and contaminants on the surface of the casting membrane caused by contact with rollers during uniaxial stretching in the MD direction [34].

### 3.2. Dry Method

The dry method is also known as the melt extrusion stretching method [30]. In a dry process utilizing uniaxial stretching, polyolefin melt is extruded and casted or blown into a membrane, then annealed within the temperature range between glass transition temperature (T_g_) and T_m_ to increase crystallinity and regulate grain size [155]. Then, the melt is stretched in the MD. Specifically, the blend is melted at elevated temperatures, extruded, and crystallized under the stress field induced by extrusion. Heat treatment is applied subsequently to promote the thickening and refinement of the lamellar crystals [34]. After cold stretching, the lamellar crystals separated into microporous nuclei, followed by hot stretching (to promote microvoid growth) and transformation from lamellar clusters into fibrous crystals. The microporous structure is then fixed by heat-setting to eliminate internal stress. Note that the quality of the prefabricated membrane depends on factors encompassing molecular weight (MW) and distribution (MWD) of the polyolefin resins, as well as extrusion–casting process parameters (i.e., die temperature, tensile ratio, cooling temperature, extrusion rate, etc.) [42]. Moreover, the processing of microporous membranes is influenced by multiple factors including stretching rate and temperature, heat-setting duration and temperature, etc. Overall, the dry stretching method has proven to be a simple and highly efficient technique, yielding a porous membrane that is well-suited for high-power density batteries.

Significantly, despite the dominance of uniaxial stretching technology in the PP membrane market through the dry method, biaxial stretching of PP can be achieved by incorporating *β*-nucleated isotactic PP (*β*-iPP) as nucleating agents [157,158]. This process aids in crystal transformation and the formation of microvoids, which occur as a result of differences in density between various phases during stretching. Moreover, this technique optimizes the uniaxial stretching process and balances tensile strength in both MD and TD directions [159]. However, biaxially stretched PP membranes generated from the dry method may have some drawbacks, including excessive thickness, lack of control over pore size and porosity, unevenness, and the need to incorporate pore-forming agents [154,160], which can compromise their practical applications. Notably, a method that takes advantage of the dry method processing has been proposed, wherein the extruded PP membrane is biaxially stretched in both the MD and TD directions without incorporation of *β*-nucleation agents [34].

### 3.3. Wet Method

The wet method, also known as the TIPS method, is a technique driven by temperature changes [41,154]. This approach involves melting, swelling, and dissolution of polymers at elevated temperatures to produce a homogeneous solution [34]. In a typical wet method, PE is blended with a solvent of low MW above its T_m_, melted to form a homogenous solution, and then extruded into a casting membrane [154]. Notably, paraffin oil is the most commonly used hydrocarbon solvent for PE membranes. After cooling, phase separation occurs, including solid–liquid or liquid–liquid phase separations with a prefabricated membrane formed. It is then heated to a temperature near T_m_ and then subjected to biaxial stretching in MD and TD to ensure homogeneous molecular chain orientation. Subsequently, the diluent is extracted and recycled, resulting in the formation of a microporous membrane [28]. Note that the wet method yields a microporous membrane featuring comparable tensile strength in both MD and TD directions due to its isotropic properties. However, the anisotropic nature of PP membranes prepared by dry method uniaxial stretching generates a significant disparity in tensile strength between MD and TD, ultimately resulting in poor tear resistance.

Notably, in wet method processes, phase separation is a critical and sophisticated event that involves crystal nucleation, growth, and microcrystalline orientation, occurring after extrusion and cooling. Moreover, nucleation, growth, destruction, and reconstruction of crystals, as well as the nonlinear evolution of multi-scale structures such as lamellar crystals, amorphous regions, fibrous crystals, and microvoids occur through synchronous or asynchronous biaxial stretching [30]. Subsequently, the solvent is extracted with a suitable extractant with a low boiling point, and internal stresses are then alleviated by heat treatment, leading to the development of a cohesive microporous structure (Figure 6). Therefore, the preparation of microporous membranes is a multifaceted coupling outcome of several phases and multiple courses involving various processing parameters, dependent on multifarious factors encompassing MW, MWD, material concentration, and processing conditions including extrusion molding, casting, biaxial stretching, heat-setting, and so on [58]. Note that the predominant advantage of PE separator membranes lies in their superior mechanical strength, which is well balanced in both directions. This can be attributed to their less anisotropic pore structure, which distinguishes them from uniaxially stretched PP-based membranes [28,30].

## 4. Recent Progress in Polymer-Based Porous Separator Membranes

In recent years, significant progress has been made in the development of polymeric-based porous separator membranes for rechargeable batteries. Various preparation techniques have been established, leading to the creation of several types of separator membranes. Notably, it is worth emphasizing that polymers play a crucial role in these membranes. Typical polymeric materials for preparing porous membranes in rechargeable batteries [7], such as LIBs, include PE, PP, PVDF, PI, polyesters, PTFE, PET, PAN, cellulose, and their derivatives, as well as blends of these materials [161,162,163]. This article aims to provide a comprehensive overview of recent breakthroughs in polymeric materials for separator membranes in the LIB field (Table 1).

### 4.1. PE-Based Separator Membranes

Microporous polyolefin membranes, particularly those fabricated from PE, PP, and their blends, have emerged as the dominant separator in the secondary rechargeable battery market. In particular, UHMWPE has gained significant market share in the commercial membrane industry through polymerization using Ziegler–Natta catalysts under low-pressure conditions [164,165,166,167]. The regular −CH_2_− structure on the elongated molecular chain in UHMWPE semi-crystalline structure imparts exceptional wear resistance that increases proportionally with MW, ranging from 3 × 10^6^ Da to 6 × 10^6^ Da. Moreover, this polymer exhibits remarkable impact strength under ordinary ambient conditions and retains robust impact resistance even at low temperatures [168,169,170,171,172]. Notably, the resultant membrane possesses superior microporous structure, exceptional puncture resistance, outstanding mechanical properties, unparalleled chemical resistance, and thermal stability, as well as ultra-high wear and impact resistance [173,174,175,176,177]. Furthermore, these separators are relatively cost-effective, thus enhancing their appeal for large-scale commercial production [7].

Nevertheless, polyolefin-based membranes are hindered by poor thermal stability due to low T_m_ and combustibility. Additionally, the hydrophobic nature of these membranes can also lead to insufficient electrolyte wettability, ultimately resulting in reduced ionic conductivity. Furthermore, shrinkage of separators and subsequent melting at elevated temperatures can cause internal short circuits, potentially leading to thermal runaway and further catastrophic security hazards [23]. Herein, these constraints have compromised their application to high-performance secondary batteries. Nonetheless, commercial polyolefin-based LIB separators still predominate in the overall LIBs markets, encompassing PE, PP, and UHMWPE due to their exceptional mechanical properties, (electro)chemical stability, mature manufacturing processes, and low cost [34]. Hence, substantial advancements have been made in improving the fundamental performance and robustness of polyolefin membranes to satisfy the extensive requirement for such membranes in the commercial market. In recent years, scientists have focused primarily on functionalizing PE membranes through surface modification to enhance properties including wettability, strength, and thermal and dimensional stability, encompassing both inorganic and organic coatings [23,81,178,179]. Specifically, inorganic reinforcement materials, such as SiO_2_, Al_2_O_3_, TiO_2_, ZrO_2_ and so on, can be incorporated into the PE matrix to prevent membrane deformation under elevated temperature conditions and further enhance the safety of LIBs, due to their excellent heat resistance and function as a physical skeleton [81,173,180].

Significantly, the utilization of inorganic and organic coating techniques, including incorporation of inorganic nanoparticles and organic polar moieties, can boost hydrophilicity of the separator surfaces [181]. For example, Wang et al. [182] utilized Al_2_O_3_ and boehmite (γ-AlOOH) as ceramic coating particles that were supplemented with BYK (Beck Chemical Co., Ltd., Altena, Germany) as an aqueous assistant to fabricate PE–Al_2_O_3_ and PE–AlOOH separators. The presence of polar group (–OH) in the PE–AlOOH separator significantly enhanced the interactions between the coating layer and PE matrix, leading to increased electrolyte uptake (i.e., 187%), higher electrolyte wettability (contact angle of 5.7°), and fairly strong ionic conductivity (i.e., 1.0 mS cm^−1^). Consequently, the electrochemical efficiency of the battery has been enhanced, encompassing both its rate capability and cycling performance. Notably, the enhancement of hydrophilicity on the surface of the PE separator can also be achieved by the incorporation of organic polar groups through chemical modifications. For example, Kim et al. [183] utilized benzoyl peroxide (BPO) to chemically treat the PE separator of LIBs, which introduced oxygen-containing functional groups such as carbonyl to both the surface and interior of the membrane. Accordingly, the hydrophobic nature of the PE separator was transformed to hydrophilic, thus enhancing electrolyte wettability and reducing the internal resistance associated with the transfer of Li^+^ ions across the separator. Note that the BPO–PE separator, as prepared, demonstrated exceptional rate capacity (i.e., 86.7% at a current rate of 3.0 C) and outstanding capacity retention (i.e., 98.1%) after 70 cycles, which is superior to the pure PE separator rate capability (i.e., 78.6%) and capacity retention (i.e., 91.7%) under the same conditions. These improvements hold great potential for enhancing the overall performance and lifespan of LIBs. Moreover, the functionalization of PE-based membranes can also be accomplished by light irradiation. For example, Sheng et al. [184] employed low-dose γ radiation to activate the PE separator and subsequently grafted methyl acetate (MA) onto it under UV light for the fabrication of the PE-g-MA separator. The inclusion of MA introduced the polar ester functional group into the PE-g-MA separator, which could effectively enhance liquid electrolyte affinity. Consequently, the PE-g-MA separator demonstrated a higher transference number of Li^+^ (i.e., 0.49) than the bare PE separator (i.e., 0.29). This exemplary performance is attributed to the increased interaction between the PE-g-MA membrane and the solvent, thereby facilitating the process of Li^+^ desolvation. Furthermore, the activation energy of the PE-g-MA separator exhibited a comparatively lower value (i.e., 52.1 kJ mol^−1^) than that of the unmodified PE membrane (i.e., 56.5 kJ mol^−1^), indicative of a more facile migration of Li^+^ across the electrode/PE-g-MA interface containing liquid electrolyte.

**Figure 7:**
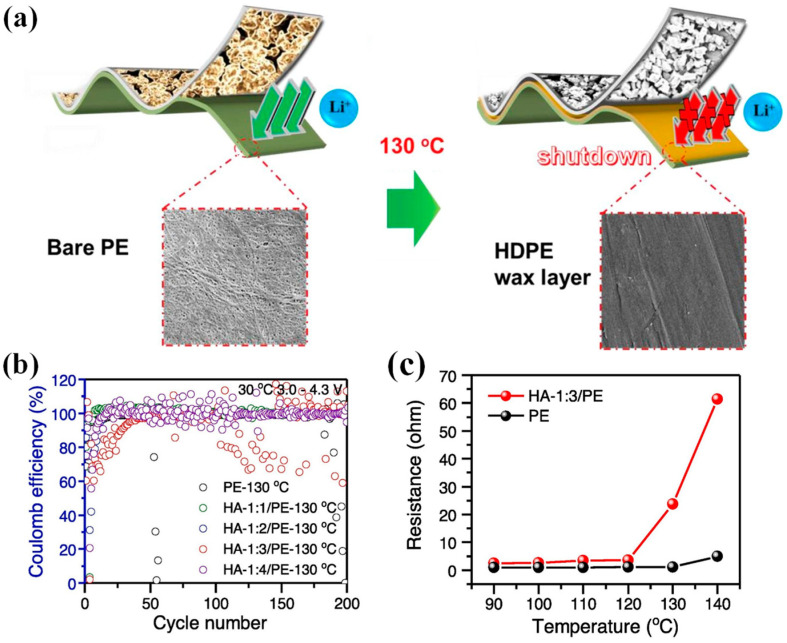
(**a**) Schematic illustration demonstrating the thermal shutdown mechanism of PE-based separator membranes with HDPE wax@AO coating layer. (**b**) Coulombic efficiency (CE) of cells with different separators heated at 130 °C. (**c**) The ohmic impedance of stainless steel (SS)||separator||SS cells with corresponding separators heated at various temperatures (90–140 °C) for 0.5 h. Reprinted with permission from reference [186], copyright Elsevier 2022.

Moreover, the core–shell structure could also be applied to enhance the performance of PE-based separators. For example, Fu et al. [185] combined SiO_2_ nanoparticles with poly(cyclotriphosphazene-co-4,4′-sulfonyldiphenol) (PZS) to prepare SiO_2_–PZS core–shell structured nanoparticles, and then coated the resulting SiO_2_–PZS on both sides of the PE microporous membrane to obtain PE–SiO_2_@PZS separator. The hydroxyl group and N, O atoms on the surface of PZS nanoparticles can act in synergy with Li^+^ ions, facilitating the dissociation of lithium salt (i.e., LiPF_6_), resulting in an increase in both ionic conductivity and discharge capacity of LIBs. Experimental findings demonstrated that the discharge capacity was 115 mAh g^−1^ at the 8 C rate, surpassing both the values of PE membranes (105 mAh g^−1^) and PE–SiO_2_ separators 106 (mAh g^−1^). Thus, SiO_2_–PZS nanoparticles characterized by a core–shell structure hold great promise as a ceramic coating material for LIBs. Xiao et al. [186] successfully developed a novel core–shell structured coating material by synergistically combining PE wax with boehmite (γ-AlOOH, abbreviated as AO), which was utilized to fabricate a high-density PE wax@AO/PE composite separator (Figure 7). The molten HDPE wax was capable of fusing and occluding pores within the separator membranes, ultimately blocking Li^+^ ion transport channels upon heating to 130 °C due to the appropriate HDPE T_m_ (i.e., 130 °C) below that of the PE substrate. By establishing a mass ratio of HDPE wax to AO of 1:3, the modified separators demonstrated superior cycling stability with 81% capacity retention over 200 cycles when compared to the unmodified PE separator, which retained only 71% capacity.

In addition to coating materials, the coating process has a significant impact on the performance of LIBs. The microstructure of the coating not only affects ion migration and electrolyte storage, but also profoundly influences the deformation and thermal shutdown of the separator when subjected to abusive conditions [187]. To achieve even coating, Wang et al. [188] employed gravure-printing method to coat pore-controllable polyamide–imide (PAI) on PE substrates. Subsequently, they prepared a series of carefully controlled PAI-coated PE separators (PAI–PE-1, PAI–PE-2, and PAI–PE-3), with pore diameters of 0.02 μm, 0.17 μm, and 0.85 μm, respectively, through a phase transfer process to eliminate nonsolvent. Significantly, the synergistic approach of combining the “guest–host transition” of PAI with the “pore on–off” mechanism of PE cooperatively provides an advanced level of security for LIBs. Compared to the severe shrinkage of bare PE membrane, the PAI layer on the PE substrate can reverse from guest to host and effectively prevent serious short circuits during overcharge, hot box, and nail test conditions. More significantly, the micropores in the PE layer exhibited a positive thermal response. Upon exposure to heat, the PE layer can constrict and close micropores, resulting in a substantial increase in its electrical resistance and forming an efficient barrier layer between the electrodes. Conversely, a battery with a bare PE separator is prone to thermal runaway as the PE material undergoes severe shrinkage and cannot effectively prevent extensive internal short circuits. Moreover, the findings indicated that, for pore sizes ranging from 0.17 to 0.85 μm, the electrochemical performance of PAI–PE-2 and PAI–PE-3 did not exhibit significant attenuation in comparison with PE membrane. Furthermore, Shin et al. [189] utilized polyethylene oxide (PEO)-functionalized ladder-like polysilsesquioxanes (LPSQ) and 4-(chloroacetyl) catechol to produce a catechol (CA)-branched organic–inorganic hybrid material (i.e., CA–PEO–LPSQ), where polycatechol and PEO were utilized as organic constituents, while LPSQ served as the inorganic component. CA–PEO–LPSQ was used as a surface modifier and coated on the PE separator through an optimized dip-coating technique at various alkaline pH levels to improve the performance of LIBs. The results indicated that the PE-M-9 separator produced at pH 9.0 exhibited excellent surface wetting properties for both aqueous solutions and battery electrolytes, with notably enhanced electrolyte absorption in comparison to unmodified PE separators. Meanwhile, the PE-M-9 separator exhibited merely 9% thermal shrinkage at 120 °C, with less deformation compared to PE-M-8 (13%), PE-M-10 (11%), and bare PE (26%) separators, thus enhancing the safety of LIBs. Furthermore, the material showed an increase in ionic conductivity (i.e., 5.79 mS cm^−1^), and the half-cell constructed using the PE-M-9 membrane demonstrated exceptional rate capability and prolonged cycle life. At 0.5 C, the capacity retention rate was 98% after the first cycle and still astonishingly high at 82% after 300 cycles.

Notably, UHMWPE has occupied a substantial market share in commercial LIB separators, owing to its superior microporous structure, puncture resistance, mechanical strength, chemical resistance, cost-effectiveness, thermal stability, and so on [173,174,175,176,177]. Just like conventional PE-based components, the performance of a UHMWPE membrane can also be enhanced when used as a battery separator through the introduction of inorganic materials (e.g., SiO_2_, Al_2_O_3_, TiO_2_, and ZrO_2_) and organic materials (e.g., PVDF, PMMA) to enhance thermal stability and improve the safety of LIBs [173,180]. As an illustration, Babiker et al. [99] efficiently engineered and industrialized UHMWPE/SiO_2_ nanocomposite membranes for the first time by means of a biaxial stretching without additional post-modifications. This novel method combined the preparation of UHMWPE separators with the functionalization of SiO_2_, effectively minimizing both costs and the duration of preparation and post-treatment procedures. When utilizing UHMWPE/SiO_2_ membranes for LIBs, the separator exhibited enhanced thermal stability with a SiO_2_ concentration of 20 wt.%. Specifically, the thermal shrinkage of MD (1.7%) and TD (1%) was significantly superior to that of a pure UHMWPE membrane, with thermal shrinkage of MD (30.5%) and TD (27.6%). Additionally, the membrane exhibited remarkable discharge capacity (i.e., 165 mAh g^−1^ at 0.1 C and 123 mAh g^−1^ at 5 C) and superior cycling performance with impressive CE (i.e., 99.93% over 100 cycles) and C-rate capability (146.2 mAh g^−1^ at current rate of 1 C). Likewise, organic compounds can also be used to enhance the performance of a UHMWPE-based membrane. Habumugisha et al. [190] employed poly(4-methyl-1-pentene) (PMP) to augment the thermal behavior and electrochemical properties of UHMWPE separators, and produced a UHMWPE/PMP blend membrane through sequential biaxial stretching. When tested on LIBs, it was found that the UHMWPE/PMP microporous separator exhibited remarkable thermal stability with a PMP content of 7.5 wt.%. After exposure to 120 °C for 1 h, dimensional shrinkage was fairly low in both MD (0.7%) and TD (1.6%). Furthermore, the membrane exhibited exceptional electrochemical performance. The rate capability test results revealed a maximum discharge capacity of 172.8 mAh g^−1^ at 0.1 C, surpassing the ideal capacity of the LiFePO_4_ cathode (i.e., 170 mAh g^−1^). Remarkably, the cells incorporating the UHMPE/PMP membrane exhibited superior and enduring cyclability, maintaining a discharge capacity of 148.0 mAh g^−1^ and a CE of 99.89%, even after undergoing 100 cycles at a rate of 1 C. Alongside enhancing battery safety by improving the thermal stability of the separator, the puncture resistance is also a crucial factor worthy of consideration. Given that any punctures produced on the membrane may potentially result in a catastrophic failure of the entire system, Li et al. [191] developed a self-reinforced composite UHMWPE membrane with nanopores (~200 nm) homogeneously embedded within interpenetrating nanofibrillar “shish kebab” networks. By maintaining molecular orientations upon pore closure, the composite material displayed a remarkable increase of 300% in both tensile strength (i.e., 550 MPa) and puncture resistance (i.e., 1.5 N μm^−1^). Additionally, cells fabricated using this UHMWPE-based separator demonstrated a 10% higher energy density and superior cyclability compared to those employing commercially available separators.

Furthermore, the performance of UHMWPE-based membranes could also be influenced by processing methodologies. Wu et al. [154] investigated the effect of wet and dry methods on the performance of UHMWPE/liquid paraffin (LP) gel membranes. The findings demonstrated that separators generated through the dry method portrayed enhanced electrochemical performance, yet produced substandard mechanical properties compared to the membrane obtained via the wet method. Therefore, to create LIB separators with optimal pore structure and exceptional properties, it is critical to merge the benefits of both dry and wet methods. Moreover, Ding et al. [192] have successfully developed a separator utilizing a wet method to create a uniform microporous structure with a porous Al_2_O_3_ nanoparticles through in situ blending, which can be easily prepared using the TIPS method involving paraffin, nano-Al_2_O_3_, and UHMWPE. This membrane has demonstrated improved electrochemical stability, ionic conductivity, and Li^+^ transference numbers due to the cooperative effect of uniformly distributed microvoids and Al_2_O_3_ nanoparticles within the porous framework, enabling facile Li^+^ transport and endowing LIBs with outstanding performance.

### 4.2. PP-Based Separator Membranes

Similar to PE, PP serves as a commonly used separator material for LIBs due to its exceptional chemical stability, thickness, and mechanical strength. However, these separators can experience significant thermal contraction at high temperatures, posing a serious threat to battery safety by potentially causing internal short circuits and leading to fire or explosion. To overcome the limitations of PP materials used in separator membranes, extensive research has been conducted in recent years, including surface modification to enhance performance, encompassing wettability, mechanical strength, puncture resistance, thermal stability, and dimensional stability with inorganic/organic coatings [23].

Yan et al. [193] employed PP as the architectural backbone to synthesize a novel multifunctional copolymer, i.e., poly(acrylonitrile-co-lithium acrylate-co-butyl acrylate) (PAAB–Li), via soap-free emulsion polymerization. Furthermore, the copolymer was utilized to create uniform-covered separators (i.e., PAAB–Li-assisted PP separators) on a PP substrate using a straightforward dip-annealing procedure. Note that the PAAB–Li-assisted PP separators exhibited superior performance, encompassing higher ionic conductivity and Li^+^ transference number value (increased from 0.360 to 0.525), as well as lower interface impedance (decreased from 155 Ω to 34 Ω) compared to the bare PP separator. Furthermore, cells containing the modified separator demonstrated consistent cycling performance for over 800 h, suggesting that the functional layer could effectively inhibit the growth of lithium dendrites. Meanwhile, LiCoO_2_/graphite cells using the as-prepared PAAB–Li-assisted separator exhibited exceptional cycle stability and rate capability. Thus, this straightforward strategy can be applied to LIBs that need high levels of safety requirements and can even be scalable to Li metal batteries. Additionally, Ding et al. [155] fabricated fumed SiO_2_/*β*-iPP membranes through the utilization of PP, silica, and *β*-nucleating agent (*β*-iPP) coupled with a sophisticated bidirectional stretching technique to optimize the porous structure and achieved fumed SiO_2_ coating in situ during the pore formation within *β*-iPP. The membrane displayed a more uniform microporous structure with a silica mass ratio of 5%, and exhibited superior discharge capacity at high current densities (67.2 mAh g^−1^ at 7 C). Moreover, the membrane maintained capacity retention above 87% even after 200 cycles, surpassing the performance of PP membranes without SiO_2_ (i.e., 70.2% after 200 cycles).

Given that short circuits may result in combustion or detonation, posing a significant safety hazard to batteries, numerous studies have focused on surface modification of membranes utilized in LIB separators in order to mitigate the negative effects of thermal shrinkage on commercial PP separators. For example, Zhao et al. [194] engineered Celgard–SiO_2_–TEOS (TEOS = tetraethylorthosilicate) separators by modifying commercial polyolefin separators (i.e., Celgard-2300) through surface chemical modification, utilizing a robust silicon–oxygen cross-linked heat-resistant network grafted onto the surface to improve membrane thermal stability. Note that the untreated separator experienced 38.6% shrinkage in the MD direction at 150 °C for 30 min, while the graft-modified separator experienced only 4.6% shrinkage. As such, these modified separators may represent a superior option than conventional silica nanoparticle-coated polyolefin separators for improving the thermal stability of LIBs. Additionally, Yu et al. [195] utilized a PI microsphere coating with high porosity and exceptional thermal stability to fabricate a PP@PI microsphere composite separator. The resulting composite separator exhibited outstanding characteristics, including thermal stability (with minimal shrinkage at 150 °C), flame retardancy, liquid retention capacity, and electrolyte wettability (with contact angles of 7° and 5°, respectively). Furthermore, the ionic conductivity is increased from 0.26 mS cm^−1^ to 0.35 mS cm^−1^. Moreover, the half-cell featuring the PP@PI microsphere composite separator boasted a high capacity of 144.3 mAh g^−1^ at 5 C, surpassing commercial PP separator-equipped battery (117.8 mAh g^−1^). Notably, LIBs equipped with PP@PI microsphere composite separators demonstrated exceptional cycle stability over 200 cycles at the current rate of 1 C. Furthermore, Chen et al. [196] have successfully developed an innovative inorganic–organic separator by directly coating the PP separator surface with zeolitic imidazolate framework (ZIF)-67 particles. Notably, two types of ZIF-67 materials, i.e., ZIF-67–H_2_O and ZIF-67–CH_3_OH, were synthesized with different solvents (i.e., H_2_O and CH_3_OH), relying on the solvent regulation effect on crystal growth to investigate the influence of ZIF-67 structures on battery electrochemical properties. Significantly, ZIF-67–CH_3_OH displayed improved cell capacity retention (61.5%) compared to the bare PP separator (0.0%) after 100 cycles at a high temperature of 55 °C. These observations suggest that LIBs’ performance is influenced by ZIF-67 samples synthesized with different solvents; hence, optimizing ZIF-67 structures could potentially enhance battery performance.

Notably, Romano et al. [197] were pioneers in synthesizing novel ultra-high molecular weight isotactic polypropylene (UHMWiPP) with reduced chain entanglements (i.e., disentangled-UHMWiPP), featuring chain disentanglement in the non-crystalline region of the semi-crystalline polymer. Note that solid-state processing of UHMWiPP opens the avenue of the preparation of disentangled molecular chains via an eco-friendly route. Note that the resultant polymers can be processed in solid state to achieve a uniaxially oriented structure below T_m_ of the nascent powder (without any solvent) to create stretched tapes boasting unparalleled mechanical properties (i.e., specific strength of 1.2 N tex^−1^, specific modulus of 28 N tex^−1^). Therefore, this innovative low-entangled UHMWiPP has demonstrated remarkable mechanical properties and superior thermal resistance to isotactic PP, making it highly promising for use as a separator material in LIBs.

Notably, there is a significant polarity disparity between the PP-based separator and the electrolyte, which poses a considerable obstacle in achieving optimal electrolyte wettability. Meanwhile, inadequate wetting ability and insufficient porosity of the separator can significantly hinder Li^+^ ion conductivity in LIBs, thus compromising their functionality and overall performance [109]. Such complications have emerged as the most significant barriers to the development of high-capacity and secure LIBs. To address this issue, Chen et al. [198] introduced bis(2,2,2-trichloroethyl) azodicarboxylate (BTCEAD) moieties onto the PP separator to synthesize PP(s)-g-BTCEAD using *N*-hydroxyphthalimide (NHPI) as catalyst. The grafted BTCEAD on the surface of PP(s)-g-BTCEAD then acted as an initiator for polymerization of poly(ethylene glycol methacrylate) (PEGMA) through activators regenerated by electron transfer atom transfer radical polymerization (ARGET–ATRP), resulting in the formation of PP(s)-g-PPEGMA with a tailored grafting degree (GD). The ionic conductivities of PP(s)-g-PPEGMA containing 8% and 18% GD were improved to 0.56 mS cm^−1^ and 0.51 mS cm^−1^, respectively. Experimental data revealed that the unmodified PP separator exhibited a rapid capacity decline after the first 100 cycles. In contrast, PP(s)-g-PPEGMA containing 18% GD could maintain stable cycling for up to 180 cycles. Hence, this work provides an effective approach for the modification of PP separators in gentle conditions with precise control over the grafting reaction. Moreover, this highly efficient tactic can be extended to modify other polyolefin separator membranes with diverse functional polymer brushes, thus achieving superior electrochemical performance for LIBs.

**Figure 8:**
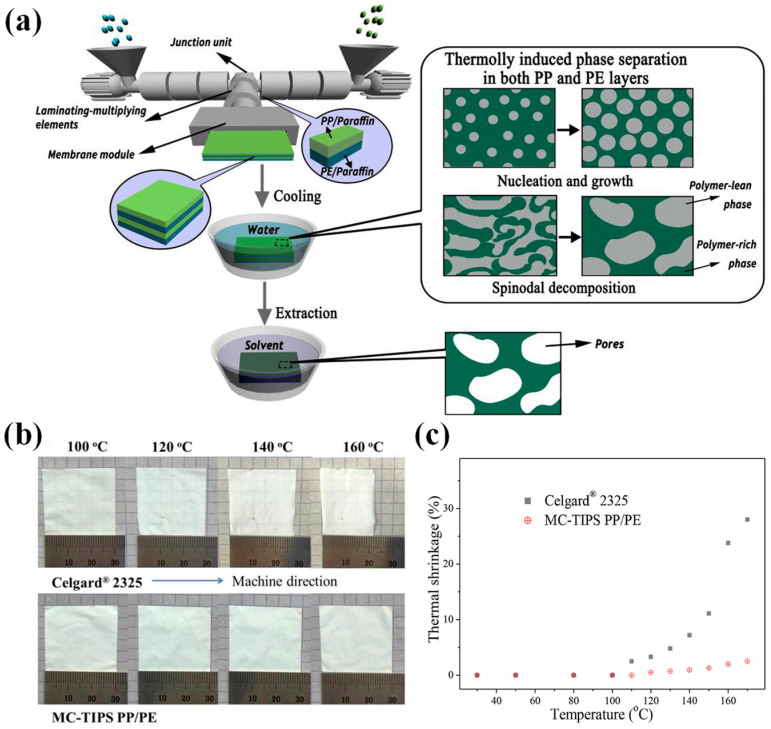
(**a**) Schematic illustration demonstrating the preparation process of MC–TIPS PP/PE separator membrane via the combination of multilayer coextrusion and TIPS method. (**b**) Typical photographs recorded for MC–TIPS PP/PE and Celgard^®^2325 membrane after heat treatment at various temperatures for 0.5 h. (**c**) Thermal shrinkage rate of MC–TIPS PP/PE and Celgard^®^2325 membrane as a function of heat treatment temperature. Reprinted with permission from reference [199], copyright Elsevier 2018.

Additionally, PP/PE multilayer separators have been commonly used in LIBs due to their superior mechanical strength and electrochemical stability. For example, Li et al. [199] employed a combination of multilayer coextrusion (MC) and TIPS methods to fabricate PP/PE multilayer separators (i.e., MC–TIPS PP/PE) (Figure 8). Note that MC–TIPS PP/PE has a cellular-like submicron pore structure and does not necessitate the cumbersome conventional stretching processes, which is advantageous for the dimensional and thermal stability of the membrane product. The dimensional shrinkage of MC–TIPS PP/PE is negligible up to a temperature of 160 °C. Furthermore, compared to commercial separators, MC–TIPS PP/PE had superior porosity (54.6%) and electrolyte wettability (i.e., electrolyte uptake: 157%, electrolyte retention: 141%), resulting in higher ionic conductivity (1.46 mS cm^−1^) and, ultimately, better battery performance. Note that this method is a one-step solution that features cost-effectiveness, environmental safety, and improved production efficiency. These remarkable merits, together with facile fabrication procedures, render MC–TIPS PP/PE a promising candidate for high-performance LIB separators. Meanwhile, in order to address the challenges associated with commercial PP-based membranes, such as lower ionic conductivity, lower porosity, and higher thermal shrinkage, Liu et al. [200] utilized a wet-laid process to modify PP with cotton fibers and PAN, resulting in a PP/PAN/cotton fiber composite membrane. Considering that cotton fibers contain a large number of polar –OH groups, while PAN fibers provide abundant hydrophilic cyano (–CN) groups, the hydrophilicity of the separator membrane is significantly enhanced. Meanwhile, PAN with high T_m_ (up to 320 °C) further contributes to enhanced thermal stability. When the cotton fiber content reached 50 wt.%, the PP/PAN/cotton fiber composite membrane demonstrated optimal overall performance, which displayed ideal tensile strength (i.e., 1.644 kN m^−1^), reasonable porosity (i.e., 63%), desirable wettability (with an aspiration height of 39 mm), and superior liquid absorption (269%). Furthermore, the thermal shrinkage of the composite membrane was below 4% following thermal treatment at 160 °C. Notably, the ionic conductivity of the fiber membrane decreased from 1.99 mS cm^−1^ to 0.32 mS cm^−1^ after 1 h of thermal treatment at 160 °C, leaving significant room for further optimization.

### 4.3. PVDF-Based Separator Membranes

Given the existing deficiencies in commercial polyolefin separators that compromise LIB applications, considerable efforts have been directed towards addressing this dilemma to improve LIB performance. Alongside techniques such as organic/inorganic composite separators and coating separators, advances in novel material technology have paved a promising avenue for enhancing LIBs. Addressing the shortcomings of existing separators is essential to the development of improved LIBs that can meet the growing demand for high-performance and environmentally friendly energy solutions [201]. Notably, PVDF has received significant attention for its potential applications in the battery industry due to its exceptional dielectric properties (i.e., ε = 8.4). Furthermore, the PVDF backbone comprises strong electron-withdrawing groups (i.e., −CF_2_−) that facilitate the dissolution of lithium salts and enhance the carrier concentration [71,123,202]. However, PVDF is a semi-crystalline polymer that may impede the absorption and swelling of the liquid electrolyte, thus diminishing the migration of Li^+^ ions, resulting in poor charge/discharge properties and inferior rate capability. To surmount these challenges facing PVDF, researchers have explored various optimization and improvement methods, including blending disparate polymers, pioneering polymer synthesis, and synthesizing composite polymer electrolytes with the incorporation of inorganic fillers.

Notably, as a hydrophilic substance, PMMA boasts excellent compatibility with liquid electrolytes and can enhance the affinity between the membrane and electrolyte, thereby improving electrolyte absorption and ionic conductivity of the separator [203]. In this regard, PMMA can be employed to blend PVDF to enhance its performance. For example, Liu et al. [204] successfully prepared a gel polymer electrolyte (GPE) by blending PVDF and PMMA via a nonsolvent-induced phase separation (NIPS) method. A satisfactory ionic conductivity (2.18 mS cm^−1^ at 26 °C) was achieved with a PVDF/PMMA weight ratio of 6:4. Moreover, the specific capacity of the cell assembled with the PVDF/PMMA membrane could retain 130.7 mAh g^−1^ even after undergoing 200 cycles at a rate of 1 C, surpassing the performance of the Celgard-2320 separator. In addition, inorganic nanoparticles can be introduced to decrease the crystallinity of PVDF. For example, Fu et al. [205] incorporated SiO_2_ nanoparticles with PMMA into PVDF membranes to create a PVDF/PMMA/SiO_2_ porous membrane. The introduction of PMMA and SiO_2_ resulted in decreased PVDF crystallinity and heightened liquid electrolyte absorption, thereby leading to improved ionic conductivity (4.0 mS cm^−1^) and reduced interfacial resistance in comparison to the Celgard separator. Moreover, the homogenous dispersion of SiO_2_ nanoparticles within the membrane can enhance the mechanical and puncture strength of separators to withstand stress and perforation, thus leading to elevated tensile strength (32.69 MPa) and high elongation at break (137.50%).

**Figure 9:**
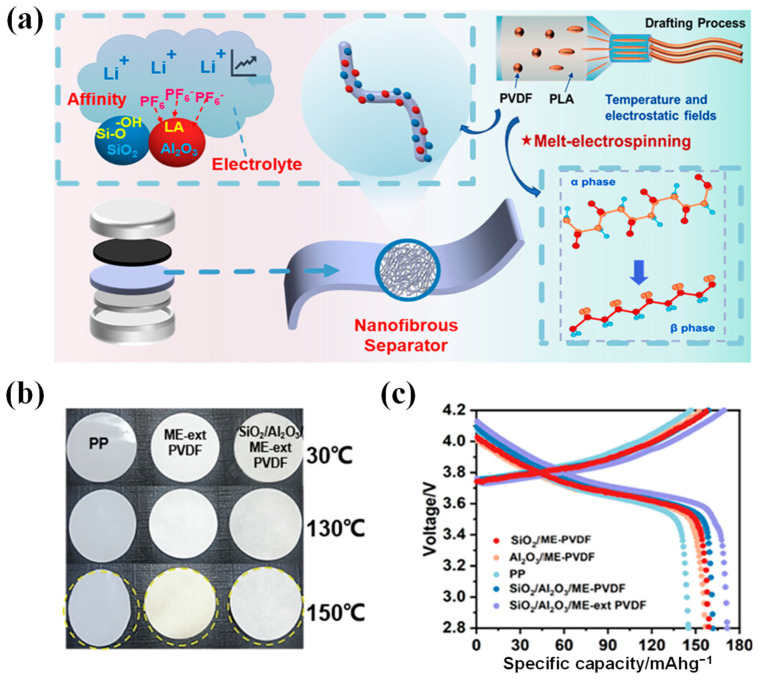
(**a**) Schematic illustration for the fabrication of the SiO_2_/Al_2_O_3_/ME-ext PVDF separator membrane. (**b**) Typical photographs recorded for PP, PVDF, and SiO_2_/Al_2_O_3_/PVDF separators after heat treatment at various temperatures for 0.5 h. (**c**) Initial charge/discharge curves of cells assembled with PP, SiO_2_/ME–PVDF, Al_2_O_3_/ME–PVDF, SiO_2_/Al_2_O_3_/ME–PVDF, and SiO_2_/Al_2_O_3_/ME-ext PVDF separators. Reprinted with permission from reference [75], copyright American Chemical Society 2023.

Although the crystalline nature has a detrimental impact on the performance of LIBs, PVDF with a *β*-crystalline phase featuring a TTTT configuration can enhance ionic conductivity. This makes it exceptionally well suited for high-performance battery separators due to PVDF’s strong polarity and dielectric properties, which result from the same dipole direction in PVDF. Therefore, Yu et al. [75] employed a novel method by combining melt electrospinning and phase-change techniques to introduce polylactic acid (PLA), a highly polar polymer, into PVDF melt for phase separation and melt electrospinning (Figure 9). Due to the robust chemical interactions between the carbonyl (−C=O) bonds in PLA and the −C−F bonds in PVDF, as well as the effects of temperature and electrostatic composite fields, there is an increase in the orderly arrangement of −C−F bonds along the macromolecular backbone of PVDF, resulting in a heightened *β*-phase of PVDF fibers. Furthermore, ceramic nanoparticles of SiO_2_ and Al_2_O_3_ were co-deposited onto the *β*-PVDF nanofiber membrane via magnetron sputtering, resulting in the formation of a SiO_2_/Al_2_O_3_/ME-ext PVDF composite separator. This innovative separator could not only enhance thermal stability, but also combat issues such as coating layer deposition and excessive thickness build-up during cycling. Moreover, the LIB equipped with this composite separator exhibited an impressive ion conductivity of 2.242 mS cm^−1^ and a high thermostability of 150 °C. Additionally, it also demonstrated excellent cycle stability and a specific capacity retention rate with a high discharge capacity (171.633 mAh g^−1^). This study proposes a feasible approach for producing an ultrafine *β*-PVDF nanofibrous separator with an ultra-thin functional coating, successfully overcoming the technical obstacles pertaining to the battery separators.

Additionally, polyvinylidene fluoride–hexafluoropropylene (PVDF–HFP) is a co-polymer of PVDF renowned for its high solvent resistance, thermoxidative degradation, and exceptional hydrophobic stability [206]. Therefore, PVDF–HFP separators are widely employed in LIB separators given their outstanding electrochemical stability, enhanced electrolyte compatibility, and increased porosity. For instance, Pinto et al. [207] fabricated PVDF–HFP through direct ink writing (DIW) by adjusting the evaporation temperature of the solvent and the percentage of fill density. They produced porous PVDF–HFP structures for the separator membranes of LIBs which displayed outstanding cycling performance and attained a maximum ionic conductivity of 3.8 mS cm^−1^ with a fill density of 100% and a solvent evaporation temperature of 25 °C. Nevertheless, PVDF–HFP separators still encounter challenges concerning inadequate mechanical strength and poor thermal stability. To address this issue, Zhao et al. [208] fabricated PVDF–HFP/TiN composite separators by incorporating TiN nanoparticles with elevated Mohs hardness (8.5) and T_m_ (2950 °C) into PVDF–HFP blends. After comprehensive characterizations and density functional theory (DFT) calculations, it was determined that the optimal amount of TiN addition was 12 wt.% under the premise of no agglomeration. As a result, TiN greatly enhanced the interaction between PVDF–HFP and the electrolyte, leading to a remarkable increase in electrolyte absorption rate (i.e., 192%). Additionally, the as-prepared membrane exhibited a high discharge specific capacity (i.e., 156 mAh g^−1^ at 1 C), exceptional cycle stability (up to 98% capacity retention after 1000 cycles), and impressive rate capability (105 mAh g^−1^ at 10 C), with a cycle life of 748 h. Furthermore, the mechanical strength of PVDF–HFP/TiN was further enhanced, as the presence of Ti-F bonds contributed to increased structural integrity. In addition, PVDF–HFP/TiN displayed exceptional thermal stability and flame retardancy.

### 4.4. Other Polymer-Based Separator Membranes

Polyurethane (PU) nanofibers have received significant attention due to their exceptional mechanical properties, heat and chemical resistance, and electrochemical stability [209]. Moreover, PU-based separators prepared by electrospinning technique exhibit great potential for flexible energy storage applications. Notably, incorporating inorganic particles into PU could enhance its electrochemical performance while simultaneously improving thermal stability. As such, Jiang et al. [210] utilized graphene and lithium acetate (LiAc) to prepare PU/G/LiAc separators. These separators can maintain original dimensions, even after being heated at 170 °C for 1 h, and exhibit high ionic conductivity of 2.47 mS cm^−1^, whereas significant thermal shrinkage was observed in the Celgard separator. Note that the PU separator containing 0.3 wt.% graphene demonstrated the highest stress of 1.67 MPa when subjected to a strain of 200%. Thermal gravimetric analysis (TGA) indicated that the weight of PU/G/LiAc materials remained unchanged until the temperature reached 300 °C. Furthermore, at current densities of 0.2, 0.5, and 1.0 C, a specific discharge capacity of 164.59, 151.95, and 132.96 mAh g^−1^, respectively, can be achieved. Compared to the Celgard separator, the testing cell with PU/G/LiAc separator exhibited excellent rate capability at a current density ranging from 0.2 to 5.0 C. The findings indicate that the freshly prepared PU/graphene separator modified with LiAc has great potential as a promising candidate for commercial polyolefin separators. In addition, in order to enhance the thermal stability of PU-based separators, Cheng et al. [211] developed a 3D composite PUC separator by electrospinning PU nanofibers filled with ceramic Al_2_O_3_ material. Compared to commercially available PP (i.e., Celgard) separators, the PUC separator demonstrated superior comprehensive performance encompassing smaller average pore size (1.088 μm), higher porosity (63.7%), greater electrolyte absorption (371%), higher ionic conductivity (6.5 × 10^−4^ S cm^−1^), and excellent thermal stability at 211 °C. Moreover, the electrode comprising the separator and LiNi_0.8_Co_0.1_Mn_0.1_O_2_ (NCM811) cathode material exhibited low interface resistance and exceptional rate capability (139.5 mAh g^−1^) at 5 C current rate, while maintaining 82.7% discharge capacity after 200 cycles at 1 C, surpassing Celgard as separator (78.8%). Overall, the battery assembled with the PUC separator demonstrated satisfactory rate capability and cycle performance, thereby presenting a novel material for LIB separators.

Notably, the PAN–PMMA membrane represents a novel separator material characterized by enhanced microstructure, porosity, and electrochemical properties. Specifically, the incorporation of PMMA enables the formation of a highly porous structure that facilitates the absorption of electrolytes into the membrane [212]. However, it should be noted that certain drawbacks, such as suboptimal wetting capability and decreased ionic conductivity, must be addressed to facilitate the deployment of this material in large-scale LIB applications. Recently, Zhang et al. [213] reported a facile method to produce PDA–PAN/PMMA membranes by surface modification of PAN/PMMA membranes using a PDA coating, which could improve the thermostability, mechanical strength, and electrochemical performance of microporous membranes. After the PDA coating, the porosity of the PAN/PMMA membrane underwent a remarkable decrease down to 56.1%, while demonstrating a significant improvement in electrolyte uptake to 421% from 365%. As the liquid electrolyte uptake increased in separators, the ionic conductivity increased from 3.20 mS cm^−1^ to 3.61 mS cm^−1^. These findings suggested that the introduction of PDA coatings could not hinder porosity of the membrane, but rather enhance its electrolyte uptake properties, possibly attributed to the adhesive performance of PDA. Note that the capacity of PDA–PAN/PMMA separator could achieve 164 mAh g^−1^ with an extremely high retention of 96.5% when the current rate returns to 0.2 C. Considering that PDA–PAN/PMMA cells exhibited reversible capacities of 162.3 mAh g^−1^ after 200 cycles, obviously higher than PAN/PMMA cells (151.6 mAh g^−1^) and PP cells (147.6 mAh g^−1^), the PDA coatings thus demonstrated enhanced cycling stability and a greater reversible capacity compared to the battery equipped with the PAN/PMMA membrane separator and commercially available PP separator.

Significantly, the prominent drawback of commercial separators is inadequate thermal stability due to their low T_m_, e.g., ~135 °C for PE and ~165 °C for PP [214]. However, polyimide, i.e., PI, is a novel type of insulating material that has gained widespread applications in various fields owing to its exceptional thermal and chemical stability properties. Remarkably, PI fulfills almost all the requirements of the LIB separator and holds great potential as the ideal separator for safe, high-voltage, high-power LIBs. To prepare the PI-based separator, Li et al. [215] utilized a NIPS method with two porogens, namely, dibutyl phthalate (DBP) and glycerin (Gly). Owing to the hydrogen-bonding interaction between these porogens, DBP dispersed uniformly in the PAA solution, leading to a PI separator with higher porosity and more homogeneous pore size distribution when compared to those prepared using a single porogen. The thickness of the PI separator was merely ~10.5 μm, resulting in lower interfacial resistance and higher specific capacity. The battery coin cells assembled with the PI separator displayed robust durability and could still function after being heated at 140 °C for 1 h, whereas those with the commercial PE separator could no longer charge due to the shrinkage of the PE under the same circumstances. Additionally, the separator demonstrated sufficient tensile strength of 23.7 MPa for battery manufacture and usage, and exhibited superior thermal dimensional stability, even at 180 °C.

Moreover, Zhang et al. [216] utilized a chemical modification strategy to enhance the effectiveness of natural polymer nanofiber-based separators by grafting cyanoethyl groups onto the surface of chitin nanofibers, resulting in the formation of cyanoethyl chitin nanofibers (CCN). The obtained CCN could form a dense membrane with exceptional tensile strength, as well as efficient transport of Li^+^ ions, due to the specific interaction between cyano groups and ions. Meanwhile, the CCN separator boasted tensile strength of up to 120 MPa, outperforming previously reported porous chitin nanofiber (PCN) separators (i.e., 80 MPa). Furthermore, the ionic conductivity and Li^+^ ion transference number of the CCN separators were significantly increased, reaching 0.45 mS cm^−1^ and 0.62, respectively, which were 13-fold and 2.5-fold higher than those in unmodified chitin nanofiber separators (i.e., 0.035 mS cm^−1^ and 0.25, respectively). Note that preliminary experiments indicated that the CCN–3M separator could maintain stable cell operation even at a high temperature of 120 °C. Thus, this novel chemical modification strategy presents tremendous potential to enhance other naturally occurring polymer nanofiber separators, encompassing bacterial cellulose nanofibers and lignocellulosic nanofibers. As such, it offers prospects for curbing fossil fuel consumption and promoting global sustainability, reinforcing its viability as a more sustainable solution.

**Figure 10:**
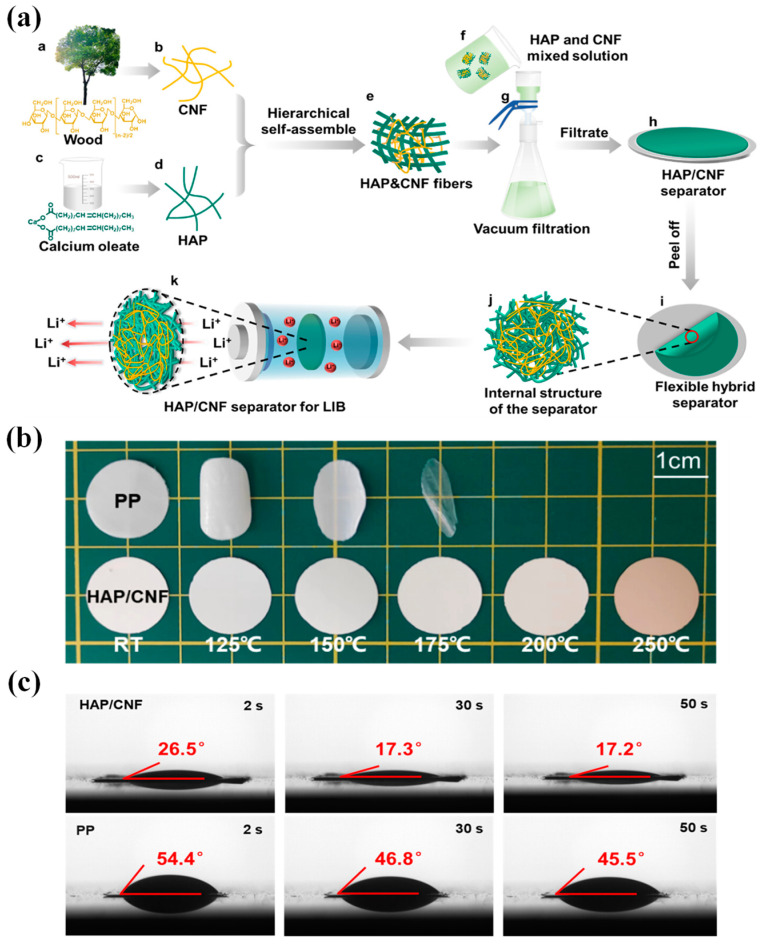
(**a**) Schematic illustration for the fabrication of hybrid porous hydroxyapatite/cellulose nanofibers (HAP/CNF) membranes and their application in LIB separators. (**b**) Typical photographs recorded for the thermal stability of separator membranes at various temperatures (upper: PP; bottom: HAP/CNF). (**c**) Contact angles recorded for electrolytes deposited on HAP/CNF and PP membranes, respectively. Reprinted with permission from reference [217], copyright American Chemical Society 2023.

Additionally, Liu et al. [217] utilized hydroxyapatite (HAP) and cellulose nanofibers, i.e., CNF, as raw materials to fabricate a HAP/CNF hybrid separator membrane (Figure 10). Compared to PP, the electrolyte wettability is significantly enhanced with HAP/CNF separators, resulting in a twofold increment in ionic conductivity. Meanwhile, the raw materials used were eco-friendly and the manufacturing processes were void of any organic solvents. Remarkably, the separator membrane showcased exceptional thermal stability (up to 250 °C) and flame retardancy. In comparison to PP separators, the HAP/CNF separator exhibited a markedly decreased contact angle with electrolytes (i.e., 17.2°), making it superior to commercial PP membranes (i.e., 45.5°). Notably, the hybrid HAP/CNF separator demonstrated a significantly higher electrolyte absorption rate of 162% compared to commercial PP-based membrane (78%), and displayed significantly improved surface smoothness with a value of 68.0 nm. Additionally, cells with the HAP/CNF membrane exhibited significantly higher capacity retention at 2 C (67.1%) when compared to that with the PP membrane (57.8%), which can be attributed to enhanced electrolyte wettability and higher Li^+^ transference numbers of HAP/CNF. Note that the capacity of the HAP/CNF separator could be maintained even after 100 cycles. Overall, the findings demonstrated that LIBs equipped with HAP/CNF hybrid separators outperformed those utilizing commercial PP separators, suggesting that HAP/CNF hybrid separators are feasible and promising in next-generation high-safety and high-performance LIBs.

**Table 1:** Summary of polymer-based separator membranes discussed in this review.

Substrate Material	Additives	Thickness (μm)	Porosity (%)	Thermal Shrinkage (%)	Electrolyte Uptake (%)	Ionic Conductivity (mS cm^−1^)	Battery Performance	Ref.
PE	Al_2_O_3_	26	55.20	0% at 130 °C for 0.5 h	144	0.75	68.4% (89.3 mAh g^−1^) after 200 cycles at 1 C	[182]
PE	γ-AlOOH	26	55.63	0% at 170 °C for 0.5 h	187	1.00	75.1% (95.1 mAh g^−1^) after 200 cycles at 1 C	[182]
PE	BPO				85	0.89	98.1% after 70 cycles	[183]
PE	MA	16				0.306	>135 cycles at 1.5 mA cm^−2^ (1 C)	[184]
PE	SiO_2_–PZS	20.4 ± 1.2			155.2 ± 14.3	1.04	115 mAh g^−1^ at 8 C; 81.69% after 100 cycles at 0.5 C	[185]
PE	HDPE wax/γ-AlOOH	10	44.0 (85 °C); 10.3 (130 °C)	<5% at 130 °C for 1 h	136.8	0.32	190.1 mAh g^−1^ (85 °C) at 0.1 C; 181.8 mAh g^−1^ (85 °C) at 0.1 C; 81% over 200 cycles	[186]
PE	PAI	14.5–15.0	35–55	1.7% (MD) and 1.1% (TD) at 130 °C for 0.5 h			98.8–99.5% after 10 cycles at 0 °C	[188]
PE	CA–PEO–LPSQ	26 ± 1.5		9% at 120 °C for 1 h	185	5.79	82% after 300 cycles at 0.5 C	[189]
UHMWPE	SiO_2_	500		1.7% (MD) and 1% (TD) at 120 °C for 1 h	472 ± 11.5	3.38 ± 0.62	165 mAh g^−1^ at 0.1 C; 123 mAh g^−1^ at 5 C; 99.93% after 300 cycles at 0.5 C	[99]
UHMWPE	PMP		65.4 ± 1.1	0.7% (MD) and 1.6% (TD) at 120 °C for 1 h	259.7%	1.17	172.8 mAh g^−1^ at 0.1 C; 99.89% after 100 cycles at 1 C	[190]
UHMWPE		2	78.3					[191]
UHMWPE	liquid paraffin	12/5	94/30					[154]
UHMWPE	nano-Al_2_O_3_	15.3	44.2	14.3% at 120 °C and 34.5% at 130 °C for 0.5 h	129.8	0.94	139.4 mAh g^−1^ at 0.2 C; 89.4% after 200 cycles at 1 C	[192]
PP	PAAB–Li	24	61.8		195.8	0.96	119.4 mAh g^−1^ at 1 C; 78.0% after 250 cycles at 1 C	[193]
PP	SiO_2_/*β*-iPP	18	42		113.7	0.82	67.2 mAh g^−1^ at 7 C; 87% after 200 cycles	[155]
PP	SiO_2_–TEOS	25.6		4.6% at 150 °C for 0.5 h		0.16	140 mAh g^−1^ at 0.2 C; 96.14% after 65 cycles at 0.2 C	[194]
PP	PI	24	54.78	0% at 150 °C for 0.5 h	207.62	0.35	144.3 mAh g^−1^ at 5 C; 80.1% after 200 cycles at 1 C	[195]
PP	ZIF-67–H_2_O	30	57.17	3.33% at 120 °C for 0.5 h	147	0.62	80.0% after 100 cycles at 1 C under 25 °C; 37.8% after 100 cycles at 1 C under 55 °C	[196]
PP	ZIF-67–CH_3_OH	30	52.53	3.33% at 120 °C for 0.5 h	142	0.78	86.9% after 100 cycles at 1 C under 25 °C; 61.5% after 100 cycles at 1 C under 55 °C	[196]
PP	BTCEAD		38.2 ± 1.30 (8% GD); 32.7 ± 2.24 (18% GD); 24.3 ± 1.49 (31% GD)		144 ± 5.9 (8% GD); 212 ± 4.5 (18% GD); 292 ± 9.4 (31% GD)	0.56 (8% GD); 0.51 (18% GD) 0.37 (31% GD)	84.59% after 175 cycles at 0.5 C (8% GD); 97.97% after 175 cycles at 0.5 C (18% GD)	[198]
PP/PE		25	54.6	0% at 160 °C for 0.5 h	157	1.46	98% (coulombic efficiency) after 30 cycles at 0.2 C	[199]
PP/PAN/cotton			63	<4% at 160 °C for 1 h	269	1.99	166.7 mAh g^−1^ at 1 C; 93.8% after 100 cycles at 1 C	[200]
PVDF	PMMA	70–80				2.18	133.3 mAh g^−1^ at 4 C; 130.7 mAh g^−1^ after 200 cycles at 1 C	[204]
PVDF	SiO_2_/PMMA	30	77		406	4.0	158 mAh g^−1^ after 50 cycles at 0.2 C	[205]
PVDF	PLA/SiO_2_/Al_2_O_3_	25			468.46	2.24	86.42% after 100 cycles at 0.2 C	[75]
PVDF	HFP	100–180			>300	3.8	38 mAh g^−1^ at 2 C	[207]
PVDF–HFP	TiN	25		30% at 175 °C for 1 h	192	1.02	156 mAh g^−1^ at 1 C; 98% after 1000 cycles	[208]
PU	graphene/LiAc		61	0% at 170 °C for 1 h	224.1	2.47	164.59 mAh g^−1^ at 0.2 C; 151.95 mAh g^−1^ at 0.5 C; 132.96 mAh g^−1^ at 1 C; 95% after 50 cycles at 0.5 C	[210]
PU	Al_2_O_3_	29.1	63.7	0% at 211 °C	371	0.65	139.5 mAh g^−1^ at 5 C; 82.7% after 200 cycles at 1 C	[211]
PAN/PMMA	PDA	65	56.1		421	3.61	172 mAh g^−1^ at 0.2 C; 162.3 mAh g^−1^ after 200 cycles at 0.2 C	[213]
PI	DBP/Gly	10.5	80 (LiTFSI); 76 (LiPF_6_)	0% at 180 °C for 0.5 h	200 (LiTFSI); 220 (LiPF_6_)	0.54 (LiTFSI); 0.55 (LiPF_6_)	75.2 mAh g^−1^ at 2 C; 87.2% after 100 cycles at 0.5 C	[215]
CCN		12	0.4 (CCN-0M); 1.6 (CCN-1M); 5.4 (CCN-3M); 4.6 (CCN-5M)	1.16 (CCN-0M); 0.59 (CCN-1M); 0.57 (CCN-3M); 0.58 (CCN-5M) at 170 °C for 0.5 h		0.45	100% (140 mAh g^−1^) after 49 cycles at 120 °C (CCN-3M)	[216]
HAP/CNF		28	76	0% at 250 °C for 0.5 h	162	0.81		[217]

## 5. Future Perspectives

The utilization of polyolefin porous membranes in commercial LIB materials has become widespread, and this technology has reached a level of maturity owing to its remarkable mechanical properties, chemical stability, and contribution to the circular economy. Recently, research has also been directed towards exploring other alternative materials, such as cellulose composite membranes and PVDF employed as bulk polymer in the phase inversion method, for the production of LIB membranes. Additionally, researchers should shift their focus towards elevating the tensile strength and puncture resistance of these membranes, aiming to avert internal short circuits prompted by external mechanical forces or the detrimental impact of lithium dendrites on membrane integrity. Specifically, in commercialized LIBs consisting of PE and PP microporous membranes, the separator pore size is typically below 1 μm, with porosity generally less than 50% to increase safety during usage. Herein, the development of a separator membrane with enhanced mechanical robustness and significantly improved porosity shall inevitably bestow a substantial boost on the overall performance of LIBs.

Simultaneously, the augmentation of the thermal endurance of the membrane (surpassing 165 °C) assumes paramount importance in averting the peril of thermal runaway within LIBs, particularly in the event of an internal short circuit. Hence, the reduction of the membrane shutdown temperature and the bolstering of its thermal stability are imperative for fortifying the safety of LIBs. Currently, coated (or composite) membranes are materializing as the vanguard of membrane application advancements. Specifically, the coatings adorning the membrane surface hold immense potential, as they contribute to an array of advantageous attributes, which encompass heightened membrane thermal stability, improved membrane conductivity when in contact with electrolytes, diminished internal resistance within the battery, and augmented discharge power. Moreover, these coatings can effectively impede or mitigate the deleterious process of separator oxidation, facilitate the seamless coordination with high-voltage anode operation, and protract the overall cycle life of the battery. Consequently, innovative coating materials and techniques ought to be explored beyond the conventional utilization of boehmite, PVDF, alumina, etc. Additionally, the development of a multifunctional composite separator capable of actively suppressing the growth of lithium dendrites presents itself as a viable avenue towards enhancing the safety and charge–discharge efficiency of LIBs.

Overall, persistent challenges pertaining to the unsatisfactory thermal stability of lithium battery separator membranes, insufficient shutdown functionality, and suboptimal ion conductivity present pressing areas of inquiry that necessitate meticulous analysis and dedicated investigation. Subsequent research endeavors should focus on the development of LIB separator membranes that boast a thin profile, exhibit heightened thermal sensitivity (manifesting in the ability to shut down at lower temperatures), elevated ion conductivity, and enhanced thermal dimensional stability. These advancements will effectively address existing flaws and dilemmas, thereby elevating the efficacy, safety, cycling capability, cost-effectiveness, and sustainability of LIBs. Notably, addressing environmental concerns entails not exclusively prioritizing application efficiency, but also considering the sustainability and ecological footprint of materials. The implementation of a robust and durable membrane not only demonstrates superior efficacy across numerous applications, but also lays the groundwork for a circular economy that curtails waste generation, minimizes process costs, and mitigates detrimental environmental repercussions.

## 6. Conclusions

LIBs have emerged as a prominent solution for energy storage, catering to the increasing demand for environmentally conscious products. As the global impetus towards sustainable energy gains momentum, the profound societal, economic, and environmental significance of LIBs is being increasingly acknowledged. The realm of LIB separator membranes has witnessed significant research endeavors, yielding a multitude of innovative systems aimed at enhancing separator performance, bolstering security measures, and addressing inherent limitations. The objective of this review is to furnish researchers with a comprehensive body of knowledge pertaining to battery separator membranes, encompassing performance prerequisites, functional parameters, production protocols, applications, and overall efficacy. Furthermore, it delves into the latest advancements in the realm of porous membranes, derived from polymeric materials such as PE, PP, PVDF, and so on, in terms of their preparation, design, modification, and optimization. Additionally, this article endeavors to shed light on the future trajectory of polymer-based composite membranes for LIB applications, while also contemplating prospective research avenues. The robust and durable membrane forged through these efforts has proven superior in efficacy across various applications, paving the way towards a circular economy that engenders waste reduction, diminishes process costs, and curtails environmental footprints.

In conclusion, the escalating demand for eco-friendly products has positioned LIBs at the forefront of energy storage solutions. The pivotal role played by separator membranes has necessitated substantial research efforts geared towards enhancing their performance and resolving pertinent challenges. Overall, the concrete content presented in this review serves as a valuable resource, fostering a profound comprehension of separator membranes and guiding the pursuit of optimized performance, ultimately contributing to the realization of sustainable energy solutions.

## Data Availability

Not applicable.
